# Genetic structure of coast redwood (*Sequoia sempervirens* [D. Don] Endl.) populations in and outside of the natural distribution range based on nuclear and chloroplast microsatellite markers

**DOI:** 10.1371/journal.pone.0243556

**Published:** 2020-12-11

**Authors:** Natalie Breidenbach, Oliver Gailing, Konstantin V. Krutovsky

**Affiliations:** 1 Department of Forest Genetics and Forest Tree Breeding, Georg-August University of Göttingen, Göttingen, Germany; 2 Center for Integrated Breeding Research, Georg-August University of Göttingen, Göttingen, Germany; 3 Laboratory of Population Genetics, Vavilov Institute of General Genetics, Russian Academy of Sciences, Moscow, Russia; 4 Laboratory of Forest Genomics, Genome Research and Education Center, Institute of Fundamental Biology and Biotechnology, Siberian Federal University, Krasnoyarsk, Russia; 5 Department of Ecosystem Sciences and Management, Texas A&M University, College Station, Texas, United States of America; Austrian Federal Research Centre for Forests BFW, AUSTRIA

## Abstract

Coast redwood (*Sequoia sempervirens*) naturally growing in southern Oregon and northern California is one of the few conifer tree species that are polyploid. Despite its unique ecological and economic importance, its population genetic structure is still insufficiently studied. To obtain additional data on its population genetic structure we genotyped 317 samples collected from populations in California (data set C) and 144 trees growing in a provenance trial in France (data set F) using 12 nuclear (five random nuclear genomic nSSRs and seven expressed sequence tag EST-SSRs) and six chloroplast (cpSSRs) microsatellite or simple sequence repeat (SSR) markers, respectively. These data sets were also used as reference to infer the origin of 147 coast redwood trees growing in Germany (data set G). Coast redwood was introduced to Europe, including Germany as an ornamental species, decades ago. Due to its fast growth and high timber quality, it could be considered as a potential commercial timber species, especially in perspective to climate warming that makes more regions in Germany suitable for its growing. The well performing trees in colder Germany could be potential frost resistant genotypes, but their genetic properties and origin are mostly unknown. Within the natural range in southern Oregon and northern California, only two relatively weak clusters were identified, one northern and one southern, separated by the San Francisco Bay. High genetic diversity, but low differentiation was found based on the 12 nuclear SSR markers for all three data sets F, C and G. We found that investigated 147 German trees represented only 37 different genotypes. They showed genetic diversity at the level less than diversity observed within the natural range in the northern or southern cluster, but more similar to the diversity observed in the southern cluster. It was difficult to assign German trees to the original single native populations using the six cpSSR markers, but rather to either the northern or southern cluster. The high number of haplotypes found in the data sets based on six cpSSR markers and low genetic differentiation based on 12 nuclear SSRs found in this study helps us study and better understand population genetic structure of this complex polyploid tree and supports the selection of potential genotypes for German forestry.

## Introduction

Although climate change can negatively affect the growth of certain native species in Central Europe [1], increased precipitation and temperature in winter expected in some regions in Germany [2] can make the environment more suitable for growing certain non-native tree species in these regions. Considering climate change and the forecast for future climate, German forestry would benefit from new adaptive management strategies [3]. To secure German timber production and the growing demand for wood products in Europe, more non-native tree species should be tested for potential introduction. One of the successful examples is the North American species Douglas-fir (*Pseudotsuga menziesii* [Mirb.] Franco), which is widely established in European forests [4,5]. The predicted altitudinal shift in the distribution of woody species [1,6] due to climate change would make Germany more suitable for species adapted to the Mediterranean climate, such as coast redwood that has already been introduced to Europe as an exotic species [7].

Coast redwood (*Sequoia sempervirens* [D. Don] Endl.) is one of the four redwood species in the Cupressaceae family [8]. All four are characterized by a particular red coloured wood, an endemic narrow natural distribution range, and are listed as threatened or endangered by the International Union for Conservation of Nature (IUCN) [8,9]. Coast redwood stands out from the other three redwood species due to its valuable wood, high growth rate and natural vegetative reproduction [10–12]. In the natural range, the western USA, it is a very important timber species growing along the Pacific Coast in southern Oregon and northern California [10]. Moreover, coast redwood was introduced into several countries with similar climatic conditions for timber production, such as France [13], New Zealand [14] and China [15].

The coast redwood natural distribution range includes the three USDA plant hardiness zones 9b, 10a and 10b (minimum temperature from -4°C to 5°C) categorized as humid Mediterranean climate [16]. Therefore, coast redwood usually experiences light frost only few weeks during a year [8] and is frost sensitive, especially at young age [10]. Its needles are able to uptake water directly from the atmosphere [17], and the tree highly depends on frequent fogs that compensate the low precipitation during summer months [10,18]. Plant hardiness zones in Germany range from 7a (-17°C to -15°C) to 9a (-6°C to -4°C) [19] and currently present a high risk for planting coast redwood for timber production in these regions. The hardiness zones 8b (-12°C to -9°C) and 8a (-9°C to -6°C) along the Rhine valley in western Germany have climate conditions most similar to the natural distribution range of coast redwood, therefore most of the planted trees can be found there. To mitigate losses in German and European forests due to climate change, it is suggested to plant adapted genotypes of major timber species, native and introduced, which are able to cope with extreme weather conditions [13]. In the case of coast redwood some trees planted in the Rhine valley exhibit potential frost resistance and survived temperatures as low as -20°C [7], but their origins are unknown. Information about their origins with the particular climatic conditions would help to select additional suitable genotypes to increase the genetic diversity of German frost resistant stands. This would improve the adaptive potential and therefore decreases risks of coast redwood timber production in Germany considering future climatic uncertainties. To achieve this goal, the current genetic diversity of German stands need to be assessed and compared to diversity of populations within the natural distribution range. Identifying distinct populations within the natural range by analysing the population structure with genetic markers will be the basis to assess their different adaptive potential. Additionally, these markers could be very efficient to assign introduced plants to their origin [4,20,21], but the detection power depends much on population differentiation [21,22]. Until now, previous coast redwood studies were not able to accomplish that based on the available allozyme and microsatellite markers [23–31].

The earlier published results based on samples from the natural distribution range genotyped using one chloroplast [30] and six nuclear [31] microsatellite markers suggested high genetic diversity, but low population differentiation with a weak indication of two clusters. The weak population structure is not surprising for a long-living, wind-pollinated tree species with relatively small area and no physical barriers for gene flow. We anticipated that additional seven expressed sequence tag SSR (EST-SSR) and six chloroplast SSR (cpSSR) markers combined with the already available five nSSRs can enhance the population structure resolution power and help us identify divergent populations. This is the first study of coast redwood where a combination of EST-SSR, nSSR and cpSSR markers was used. In contrast to the random nuclear nSSRs, EST-SSR markers could be potentially under selection [32] and, although cpSSRs are more conservative and have a lower mutation rate than random nuclear nSSRs, they could be also very polymorphic and informative [33], especially for tracing long distance migration because of their strict paternal inheritance via pollen in coast redwood [34]. In our study we used already published five microsatellite or nuclear simple sequence repeat (nSSR) markers [29,31,35] together with newly developed six cpSSR and seven EST-SSR [36] markers. This is also the first study presenting data on SSR markers genotyped in both Californian and German coast redwood populations.

We expected that this new marker combination would improve the resolution of population structure within the natural distribution range and help us discriminate populations. In case of highly differentiated population structure we should be able to assign German frost resistant trees to their origin. In addition to the samples representing the complete natural distribution range, we collected samples from sites with suboptimal growing conditions in California and included them in our analysis hoping that they could help us identify the origin of German trees that are supposedly adapted to drier and colder climate and could likely originate from sites with colder and drier environment. The obtained data can help to select the most appropriate genotypes for *in-situ* conservation management strategies and timber production in and outside the natural distribution range.

The main objectives of this study were to 1) compare genetic population structure resolution based on nuclear (EST-SSRs together with nSSRs) vs. chloroplast (cpSSRs) microsatellite markers, 2) study genetic diversity based on EST-SSRs, nSSRs and cpSSRs within and outside the natural distribution range, and 3) assign trees growing in Germany to the original populations.

## Materials and methods

### Plant material

**Fig 1:**
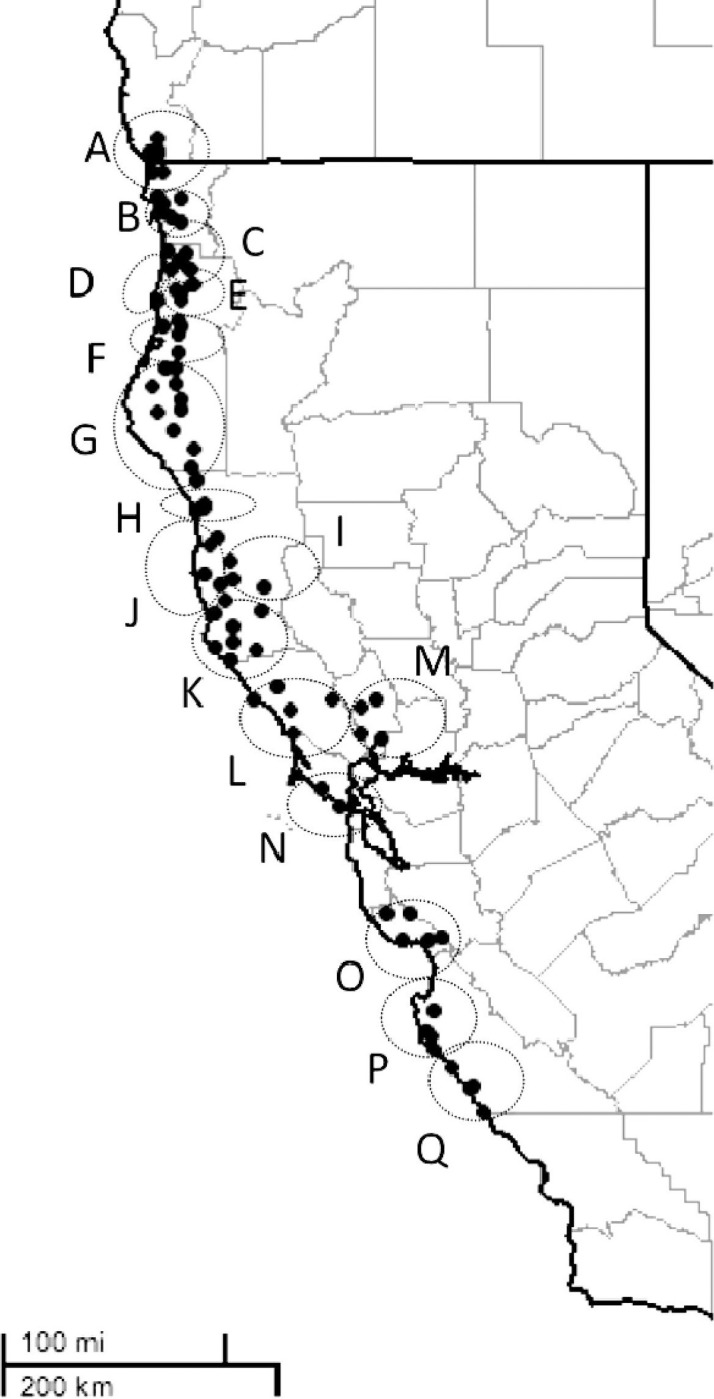
Map of 17 watersheds (A-Q) along a latitudinal range from 42° 12' to 35° 55' N within the natural distribution range of *Sequoia sempervirens* for the French data set F (Fig 1 in Douhovnikoff and Dodd [31] reproduced with permission from The American Midland Naturalist).

Three data sets were used in this study: French (F), Californian (C) and German (G). In 1984 an international provenance test was established by Kuser et al. [37] (so-called “Kuser provenance test”) using seedlings representing 90 provenances in the natural distribution range of coast redwood (S1 Table). The 180 individuals from this test were propagated by cuttings and used to establish hedge orchards in California, Oregon, Spain, Britain, France and New Zealand, including a planting site established in 1990 in St. Fargeau, Central France. For data set F, depending on accessibility, either needle or cambium (when braches with needles were unreachable) material was collected in 2014 from 153 surviving trees in St. Fargeau and frozen until DNA extraction. The data set F was compared with another “Kuser provenance test” experiment established in 1984 at a site close to Berkeley, California, named the Russell Reserve. Douhovnikoff and Dodd [31] partitioned 135 Russell Reserve trees into 17 watersheds according to their original geographic location and the GPS data (Fig 1) for population genetic structure analysis based on six nSSR markers [31]. To compare our data with their results and population based analyses [31], the St. Fargeau samples in the data set F were also partitioned into the same 17 watersheds also based on their original geographic location (S2 Table). Original locations of the ‘Kuser’ samples placed on the map with the mean monthly temperature pattern for the time period 1979–2013 are presented in S1 Fig.

**Fig 2:**
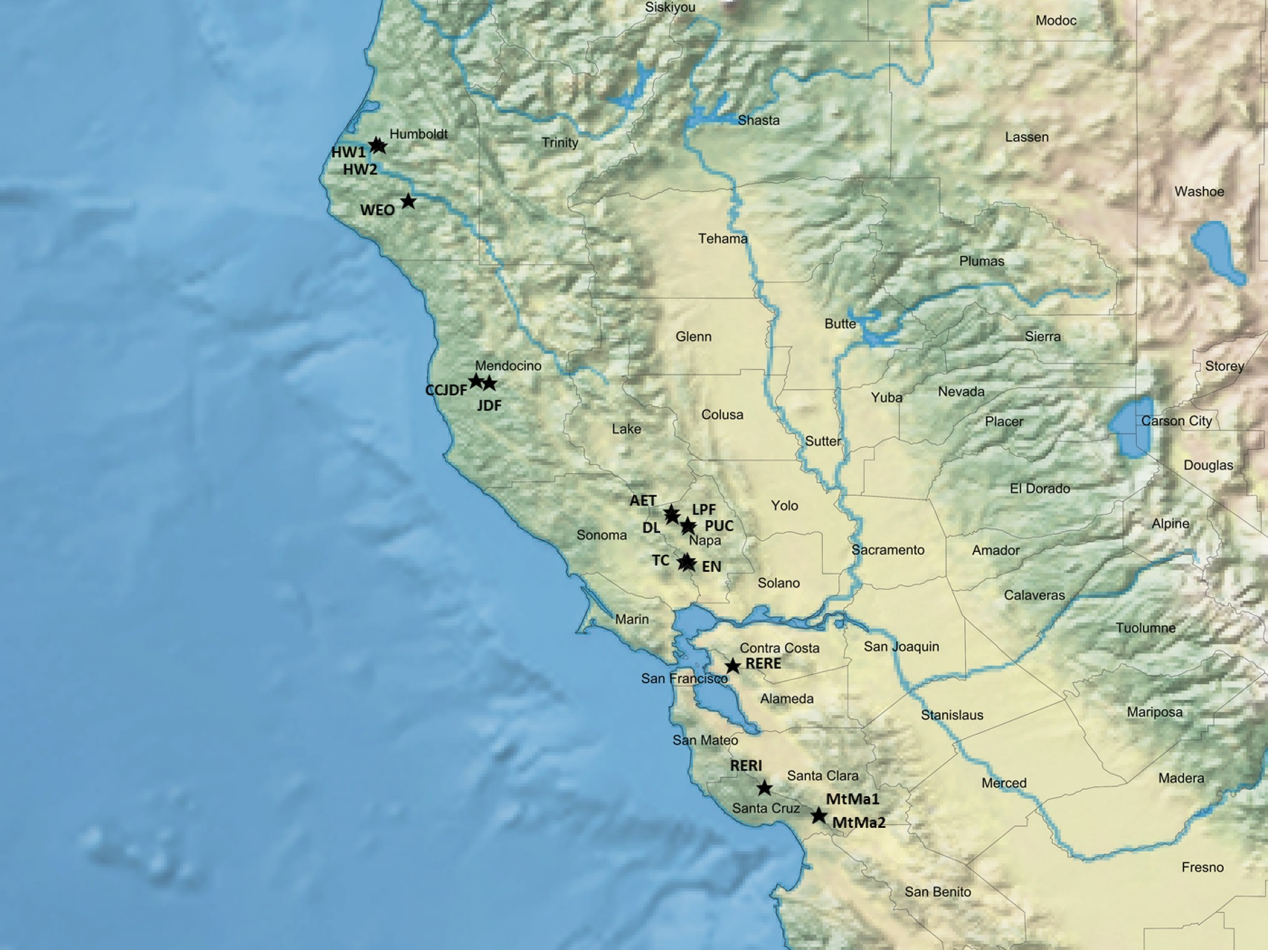
Collection sites for the Californian data set C depicted by stars in the California map generated using the SimpleMappr online software (https://www.simplemappr.net).

The Californian data set C consisted of 309 tree samples collected from 16 locations within the natural distribution range in central and northern California in 2017. Number of samples, altitude, historic precipitation and mean January temperature per location are listed in S3 Table. The locations were chosen according to their geographic position and climatic conditions. The optimal climatic conditions for coast redwood are along the coast, with high oceanic influence and humidity and high ground water level [38]. The locations selected for sampling were at higher altitude, more interior, with colder and less foggy conditions, and therefore represented drier and more extreme habitats [18,39]. The original locations of 12 frost resistant trees in the St. Fargeau provenance site were also considered while selecting sources for the potentially frost resistant trees. These 12 frost resistant trees were identified by the owner in 1992, after a late frost occurrence in 1991 (unpublished data). Among the selected sites it was possible to visit 16 locations (Fig 2), and trees with a minimum diameter at breast height (DBH) of 15 cm were randomly sampled at each location. It should be noticed that there is no information proving that the sampled California stands represented natural populations. Some or many of them could be at least managed, if not even planted. The collected needle material was dried and stored in silica gel until DNA extraction.

The German data set G included in total 147 samples representing six sites: “Sequoiafarm Kaldenkirchen”, “Aboretum Burgholz”, “Weltwald Bad Grund” and three German Botanic Gardens in Bayreuth, Chemnitz and Göttingen, respectively (S2 Fig and S4 Table). The “Sequoiafarm Kaldenkirchen” was established by the Martin family in the 1950’s using coast redwood seedlings from probably one single tree in the Californian “Schenck-Grove”, Prairie Creek Redwoods State Park (Humboldt County, California, USA). Cuttings from these “best performing” clones were planted all over Germany and are known as the “Martin-Clones” (M. Geller, pers. communication). More than 100 individuals were planted in the “Aboretum Burgholz” representing the “Martin-Clones” and other sources in unknown numbers and combinations. The “Sequoiafarm Kaldenkirchen” and “Aboretum Burgholz” are both located in the Rhine valley in the plant hardiness zones 8a and 8b [19], respectively. The “Weltwald Bad Grund” is an arboretum located in the western Harz Mountains near Bad Grund with relatively cold winters and dry summers, typical for the plant hardiness zone 7b. Coast redwood cuttings and possibly seedlings from trees of the “Aboretum Burgholz”were planted in the Weltwald, but also in unknown number and combinations. Coast redwood trees collected from botanic gardens in Chemnitz, Bayreuth and Göttingen represented few individuals with unknown origins that survived in the coldest areas in Germany. Chemnitz and Bayreuth are in the plant hardiness zone 7a, and Göttingen in zone 7b. Fresh needle material was collected from all these individual trees in Germany and frozen at -20°C until DNA extraction.

For allele scoring validation, needles from 30 approximately three-year-old clones, including 2–3 ramets per clone, respectively, were collected in the Allerweltsgrün nursery (Köln) and frozen at -20°C (S3 Fig).

### DNA isolation and PCR amplification of the microsatellite (SSR) markers

DNA was isolated from needles or cambium using the DNeasy Plant Kit (Qiagen, Hilden, Germany) following the manufacturer’s instructions. The isolated DNA was diluted in ddH_2_O 1:10 for PCR amplification and stored at -20°C.

Five nSSR, seven EST-SSR and six chloroplast (cpSSR) microsatellite markers were used in this study to genotype samples in all three data sets F, C and G, respectively (S5 Table). The same touch-down PCR program was used for all 18 PCR primer pairs following the protocol described in Breidenbach et al. [36]. The PCR products were separated and visualized using the ABI Genetic Analyser 3130xl with GENSCAN ROX 500 as an internal size standard.

### Verification of the nuclear microsatellite markers

The PCR primer nucleotide sequences for the 12 nuclear SSR markers were mapped to the coast redwood draft nuclear and chloroplast genome assemblies (made recently publicly available) to verify annealing sites for the microsatellite markers used in this study. The primer sequences were mapped against scaffolds downloaded from the NCBI GenBank (accession number VDFB00000000) using the CLC Genomic Workbench v11.0.1 software (Qiagen, Hilden, Germany) with the following parameters: complete match for the last 15 nucleotides at 3’ end, maximum mismatch for 3 nucleotides per annealing site in total and a maximum of 400 nucleotides between annealing sites.

### Genotyping of the nuclear microsatellite markers

GeneMapper 4.1. (Thermo Fisher Scientific, USA) was used for visualization and fragment size calling of the PCR products. Coast redwood is a hexaploid species [40], which complicates microsatellite scoring [35,41]. In this study, we used the genotyping method of Pfeiffer et al. [42] to identify consistent and reproducible alleles. Following this method, all fluorescent peaks detected in 2–3 ramets per each of 30 different clones and representing different PCR amplified fragments were assigned in each sample to one of the 10 rank categories according to the shape and intensity of the peak and the motif of the primer. To determine which ranking has the highest probability, input files with different ranking combinations from category 7 to 10 with all 12 primers were generated. For example, for the ranking combination 7, all peaks of each sample assigned to the category 7 and above (8, 9 and 10) were included in the analysis, for the ranking combination 8, all peaks of each sample assigned to the category 8 and above (9 and 10) were included into the analysis. The R-package “polysat” [43,44] specifically developed to study genetic data of polyploid species was used for each ranking combination data set to calculate the pairwise ‘*bruvo genetic distance’* between individuals [45]. The supposedly compound RW56 marker, in fact, demonstrated alleles which sizes followed regular tetranucleotide repeat differences in our study. Therefore, it was treated as a regular SSR marker with a tetranucleotide motif.

Using the R-package “ape” [46] and the pairwise distance matrix, a neighbour-joining (NJ) consensus tree was generated based on 1000 bootstraps. Since each marker differed in the allele pattern and peak shape, 25 different ranking combinations for the markers were tested. The ranking combination with the highest bootstrap value and correct grouping of the ramets representing the same clone was used for all further genotype scoring. The NJ results were confirmed with the function “assignClones” of the R-package “polysat” [43] using the miss-matching threshold of 0.2. The markers final ranking categories were from 7 to 10 (S5 Table). The NJ tree (NJT) based on the final ranking combination of the markers and 1000 bootstraps is presented in S3 Fig. In addition, for the data sets F and C, scored genotypes were converted also into a presence-absence matrix, and the Nei’s genetic distances ([47] after [48]) were calculated between watersheds F and populations C using the AFLP-SURV software with 1000 permutations [49]. The genetic distance matrix was used to generate a NJT with the PHYLIP v.3.69 software [50], respectively. The consensus tree was visualized using the FIGTREE software [51]. NJTs, using cpSSRs, were based on Nei’s genetic distance ([47] after [48]) and 1000 bootstraps for the reference data sets C and F and generated using the R-packages “adegenet” [52,53] and “poppr” [54,55]. To facilitate comparison between the phylogenetic trees based on the watersheds in data set F and Douhovnikoff and Dodd [31], watershed I (Mendocino County) was also used as a root of the NJT.

To compare genetic diversity levels within and outside the natural distribution range, the data sets F, C and G were also combined into one set and partitioned into the following three subgroups: NORTH and SOUTH representing the watersheds and locations located either North or South of the San Francisco Bay, respectively, and GER representing the German trees in the data set G. The R-package “polysat” was also used for the calculation of number of alleles, Shannon index as a parameter of genetic diversity and number of private alleles for each population in C, watershed in F, and group, respectively. Further, pairwise Jost’s *D* and *F*_ST_ parameters of genetic differentiation were calculated and bootstrapped 1000 times to identify significant values using the R-package “polysat”. Additionally, analysis of molecular variance (AMOVA) was conducted for data sets C, F and G with 999 permutations, respectively using the converted presence-absence matrix and the software GenAlEx [56].

### STRUCTURE analysis

Based either on 12 nuclear SSR or six cpSSR markers, input files with the original allele sizes for the data sets F and C were used by the STRUCTURE v2.3.4. program [57–60] to infer the most likely potential number of distinct genetic clusters (*K*) by testing for different number of *K* from 1 to 24 with 20 iterations per run using the MCMC with 10 000 burn in’ and 100 000 final iterations assuming the admixture model [61].

The STRUCTURE HARVESTER [62] and the ClumPPAK [63] programs were used to visualize the STRUCTURE results and to help determining the most likely number of clusters using the Δ*K* approach. The STRUCTURE analyses were repeated also with the “locprior” optional parameter engaged (which is used to assist the clustering by specifying populations *a priori*), because low population differentiation was expected. Following the STRUCTURE HARVESTER results, the data sets F and C were divided each into two separately analysed groups—12 northern populations and four southern populations located to the north or south of the San Francisco Bay, respectively, to test for further clustering within these two groups using the same STRUCTURE settings.

In addition, the German sample (G) together with French (F) and Californian (C) samples was also analyzed with STRUCTURE for *K* = 17 (number of watersheds) and *K* = 30 (total number of subpopulation samples or groups in the F and C data sets) by using USEPOP flag (known origin) for F and C samples, but not for G (unknown origin) samples to assign individual German trees to either a particular watershed or a particular sample, respectively. The *Q*-values in two *Q*-matrices obtained for *K* = 17 and *K* = 30, respectively, were used as genetic traits to generate two pairwise genetic distance matrices using geometric index of genetic similarity developed by Zhivotovsky [64]. Briefly, using *Q*-matrix $Q=\left[\begin{array}{cccc} p_{11} & p_{12} & \ldots & p_{1K}\\ p_{21} & p_{22} & \ldots & p_{2K}\\ \ldots & \ldots & \ldots & \ldots \\ p_{n1} & p_{n2} & \ldots & p_{nK} \end{array}\right]$, where *n* is a total number of trees, *K*–number of clusters used by STRUCTURE to generate the *Q*-matrix, and each tree is presented by *Q*-values, so that their sum in each row equals 1: *P*_*i*1_+*P*_*i*2_+_*…*_+*P*_*iK*_=1 for any, *i* (*i*=1,2,…*n*), then, index of similarity (*r*) between trees *i* and *j* is calculated as $r_{ij}=\sqrt{p_{i1}p_{j1}}+\sqrt{p_{i2}p_{j2}}+\ldots +\sqrt{p_{iK}p_{jK}}$. This index is always positive (non-negative) and does not exceed 1. The advantage of this index is that it takes into account even small *Q*-values. When vectors (trees in our case) are completely identical, then, it is equal to 1, and when the vectors are completely orthogonal, it is 0. The distance is simply calculated as 1-*r*.

Then, the obtained distance matrices were used for cluster analysis by generating NJTs with the R-package “ape” [46] and Principle Coordinate Analysis (PCoA) plots with GenAlEx [56].

### Genotyping of the chloroplast microsatellite (cpSSR) markers and haplotype network

Due to the haploid nature of chloroplasts, genotyping of the cpSSR markers was easier and unambiguous. Based on all three data sets, a haplotype network was built using the Goldstein distance [65] in the program EDENetwork v2.28 [66]. The program calculates a weighted network based on the Goldstein distance between haplotypes with an automatically calculated percolation threshold of 2.67 [67].

### Genetic assignment using the chloroplast microsatellite (cpSSR) markers

The individuals from the German data set G were assigned to the watersheds and populations in data sets F and C using the cpSSR markers and the GeneClass2 software [68]. For data set C, eight closely located sampling sites (< 1.0 km) were combined into four: PUC and LPF were combined into LPF (watershed M), MtMa1 and MtMa2 into MtMa (watershed O), HW1 and HW2 into HW (watershed G), and JDF and CCJDF into JDF (watershed J). A quality assessment (QI) was based on the self-assignment test following Hintsteiner et al. [4] using the computation criteria of Rannala and Mountain [69], the simulation algorithm of Paetkau et al [70] with 10 000 resampled individuals, and Type 1 error of 0.01 [69,71].

## Results

Mapping of the PCR primer nucleotide sequences for the SSR markers to the draft coast redwood nuclear and chloroplast genome assemblies found annealing sites facing each other in a correct configuration allowing amplification for all six chloroplast markers and eight out of 12 nuclear markers (S7 Table). Three different single scaffolds included annealing sites for three markers *ss36782*, *ss73361* and *ss114481*, respectively, two different scaffolds included annealing sites for each of two markers *RW48* and *ss73307*, respectively, three, four and six different scaffolds included annealing sites for *ss73978*, *ss91170* and *ss74800* markers, respectively. The chloroplast markers all had only a single annealing site pair per each marker in the chloroplast genome, but multiple scaffolds also contained annealing sites. The latter could be due to either incomplete removal of chloroplast sequences from the nuclear genome assembly or presence of chloroplast sequences in the nuclear genome as a result of misassembling or migration of chloroplast genes to the nuclear genome [72–74].

**Fig 3:**
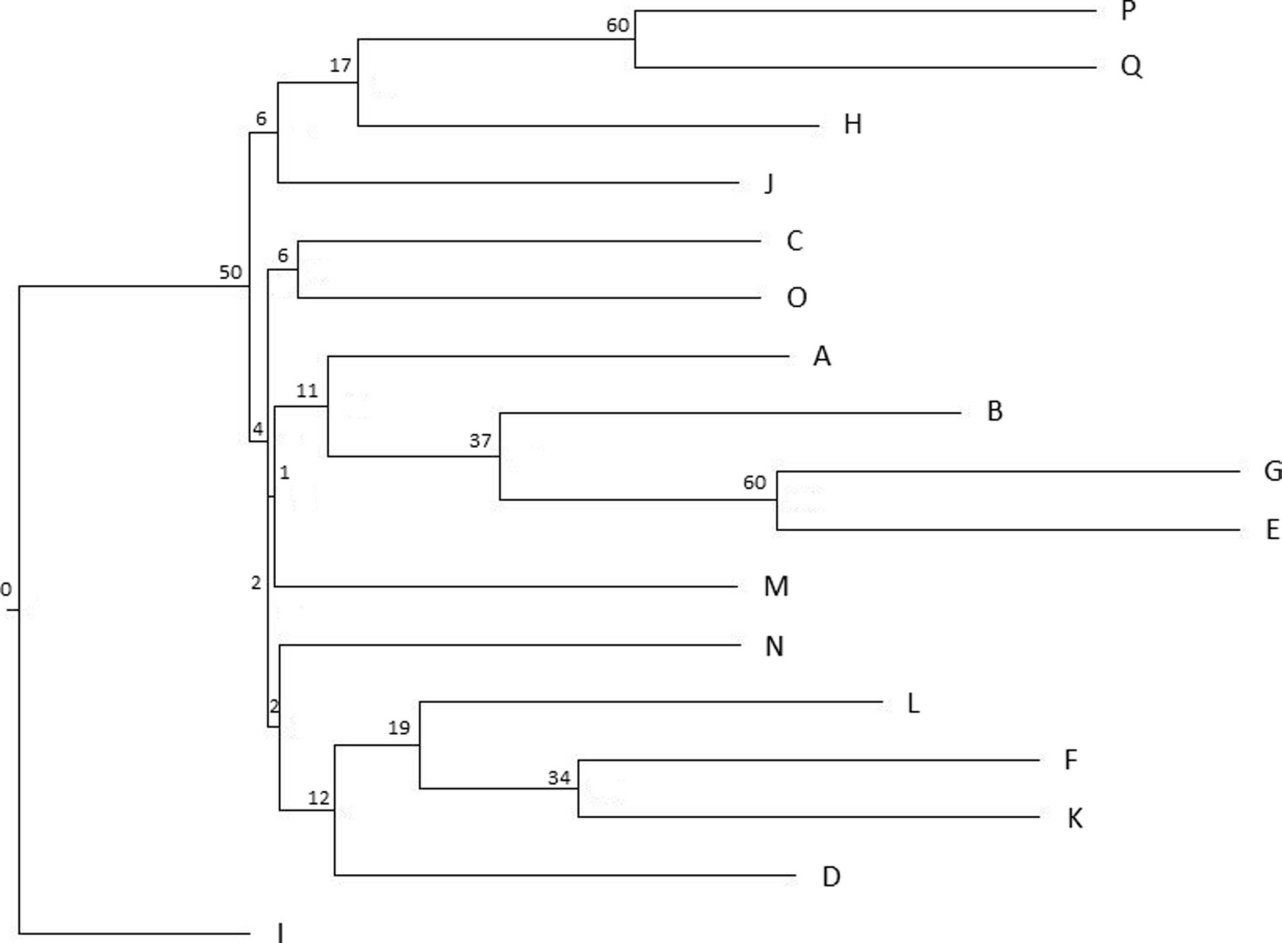
The neighbour-joining tree (NJT) for the French data set F partitioned into 17 watersheds (A-Q) following Douhovnikoff and Dodd [31] based on Nei’s genetic distance ([47] after [48]) calculated using 12 nuclear SSR markers. Watersheds from A to Q are distributed from north to south in central California (see also Fig 1 and S1 Table). Bootstrap values are presented as percentage.

**Fig 4:**
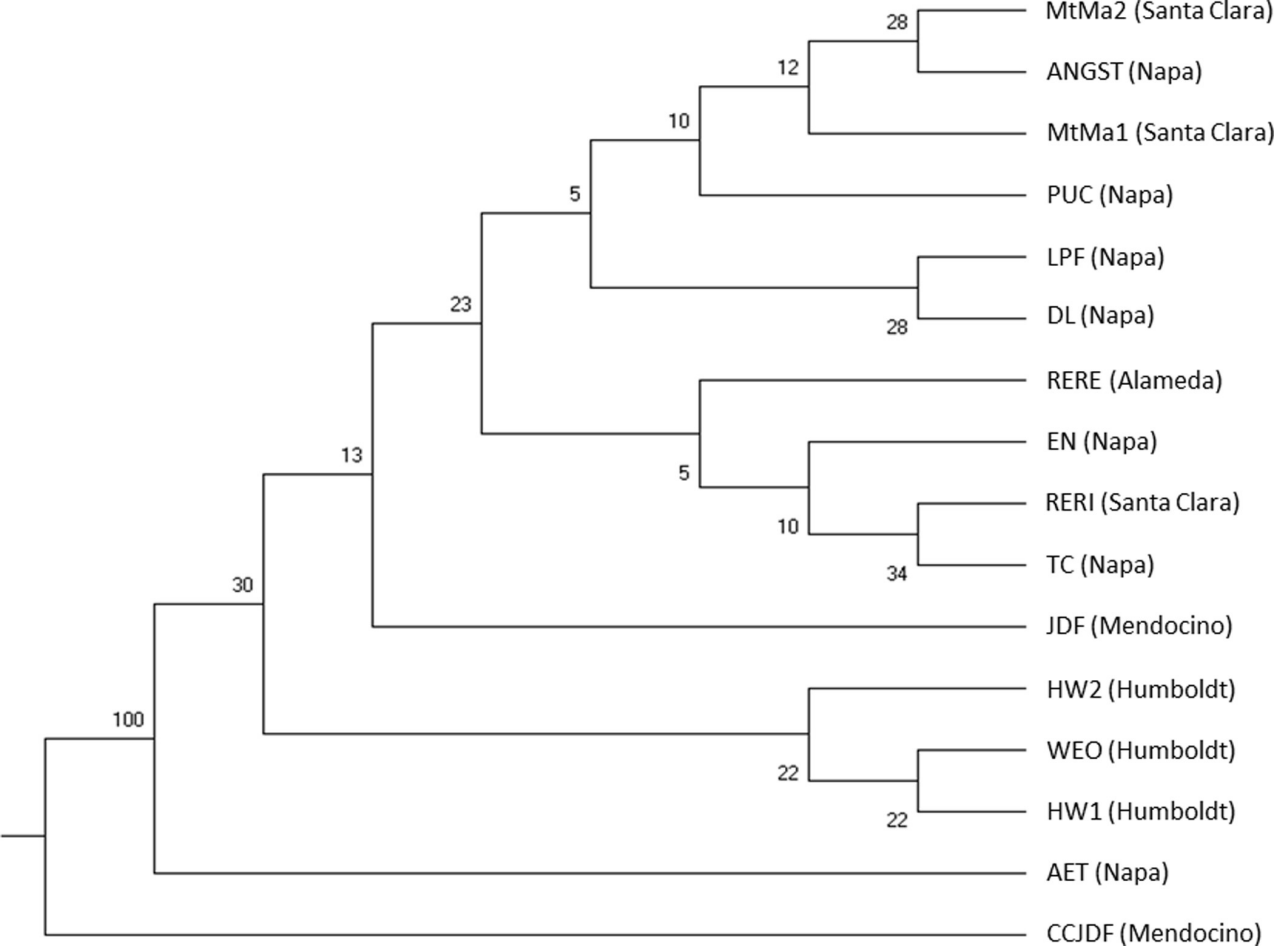
The neighbour-joining tree (NJT) for populations in the Californian data set C based on Nei’s genetic distance ([47] after [48]) calculated using 12 nuclear SSR markers. County names of the sampled locations are in brackets. Bootstrap values are presented as percentage.

The NJTs for the data sets F and C had low bootstrap values below 60% indicating weak differentiation between watersheds and populations based on 12 nuclear SSR markers (Figs 3 and 4), except for two watershed pairs P-Q and E-G in the data set F (Fig 3) and populations AET and CCJDF in the data set C (Fig 4). The NJTs based on the cpSSR markers showed higher bootstrap values for most clusters, but they did not reflect their geographic relationship (S4 and S5 Figs).

**Fig 5:**
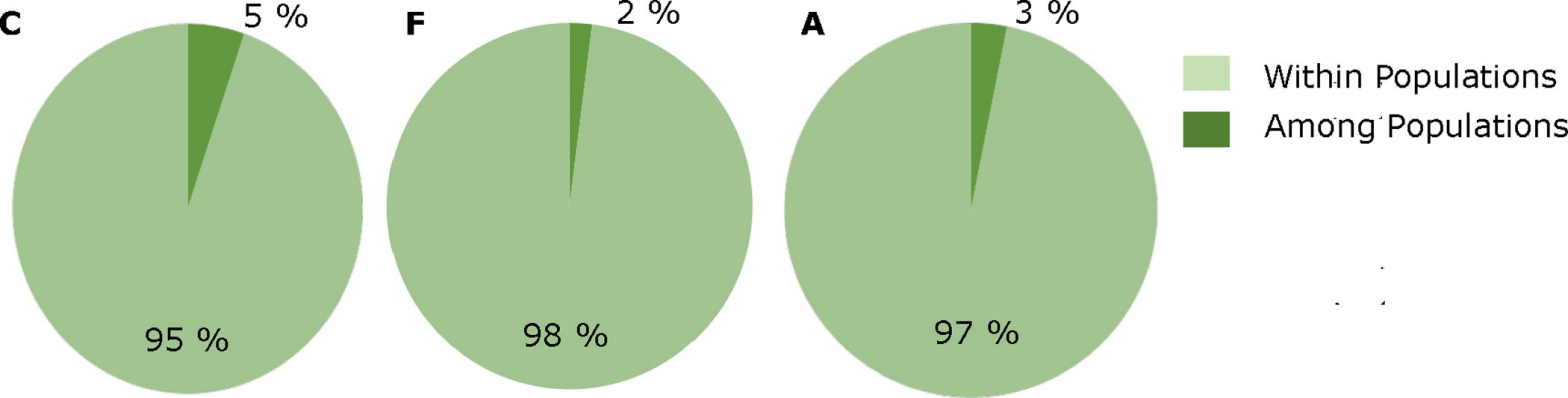
AMOVA results based on 999 permutations for data sets C and F and groups NORTH, SOUTH and GER (G) using the 12 nuclear SSR marker genotypes transformed into binary data.

Highest genetic diversity values were found in northern and southern populations HW2 and MtMa1 belonging to data set C, and in northern watersheds G and L belonging to data set F (Table 1). The highest Shannon index (5.58) was found in the group of northern populations (NORTH) and the lowest (3.76) in the group of German samples (GER). Populations and watersheds with high genetic diversity contained also high number of alleles (Table 1). Highest numbers of private alleles were found in populations AET and RERI and watersheds J and N. AMOVA results revealed high variation within populations for all three data sets C, F and G. Highest variation among populations was found for data set C (Fig 5). Pairwise Jost's *D*- and *F*_ST_-values were low and insignificant (S6 Table). Within data set C, population AET was the most and HW2 the least differentiated in comparison to all other populations, except RERI and MtMa1 (S6A Table). Within data set F the watersheds O, P and Q located to the south of the San Francisco Bay were differentiated from the watersheds B, C, D and E located to the north of the San Francisco Bay (S6B Table).

**Table 1:** Diversity measures for populations in Californian data set (C), 16 watersheds (A-Q) in French data set (F) and three groups consisting of the trees pooled from populations located above (NORTH) and below (SOUTH) San Francisco Bay in both C and F sets, and all German genotypes together (GER) based on 12 nuclear SSR markers.

Watershed	Population	Data set	N	Total number of alleles	Shannon Index	Number of private alleles
**North**						
**A**		F	8	83	2.08	3
**B**		F	8	79	2.08	4
**C**		F	7	70	1.95	2
**D**		F	5	62	1.61	1
**E**		F	5	62	1.61	1
**F**		F	8	80	2.08	1
**G**		F	13	95	2.56	1
**G**	**HW1**	C	29	158	3.14	4
**G**	**HW2**	C	47	189	3.78	5
**G**	**WEO**	C	11	117	2.16	0
**I**		F	7	79	1.95	1
**J**		F	8	85	2.08	6
**J**	**CCJDF**	C	16	128	2.52	3
**J**	**JDF**	C	13	122	2.30	3
**H**		F	6	64	1.79	1
**K**		F	8	81	2.08	3
**L**		F	9	86	2.20	5
**M**		F	5	67	1.61	3
**M**	**AET**	C	6	65	1.39	5
**M**	**ANG**	C	6	83	1.61	0
**M**	**DL**	C	15	117	2.56	3
**M**	**LPF**	C	12	126	2.48	2
**M**	**PUC**	C	26	151	3.03	5
**M**	**EN**	C	17	138	2.77	2
**M**	**TC**	C	17	136	2.77	1
**South**						
**N**		F	6	77	1.79	6
**N**	**RERE**	C	11	109	2.20	3
**O**		F	7	79	1.95	5
**O**	**RERI**	C	26	158	3.14	6
**O**	**MTMA1**	C	35	149	3.21	3
**O**	**MTMA2**	C	22	148	3.00	4
**P**		F	4	56	1.39	3
**Q**		F	4	62	1.39	1
**Groups**						
	**NORTH**	C & F	267	240	5.58	60
	**SOUTH**	C & F	86	192	4.45	21
	**GER**	G	44	152	3.76	18

**Fig 6:**
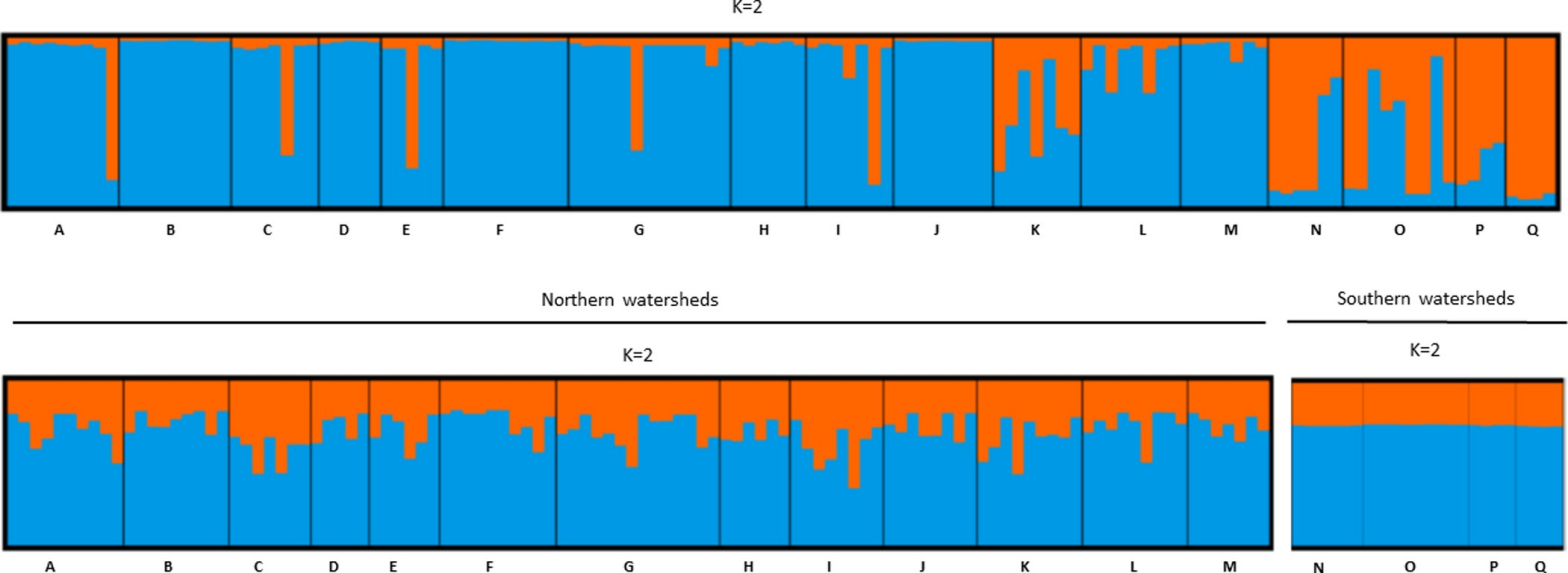
STRUCTURE analysis demonstrating the most likely number of clusters (*K*) using the “locprior” function for 16 populations in the Californian data set C based on 6 cpSSR markers and two separately analysed groups; 12 northern populations (*K* = 3) and four southern populations located to the north or south of the San Francisco Bay, respectively (*K* = 4).

**Fig 7:**
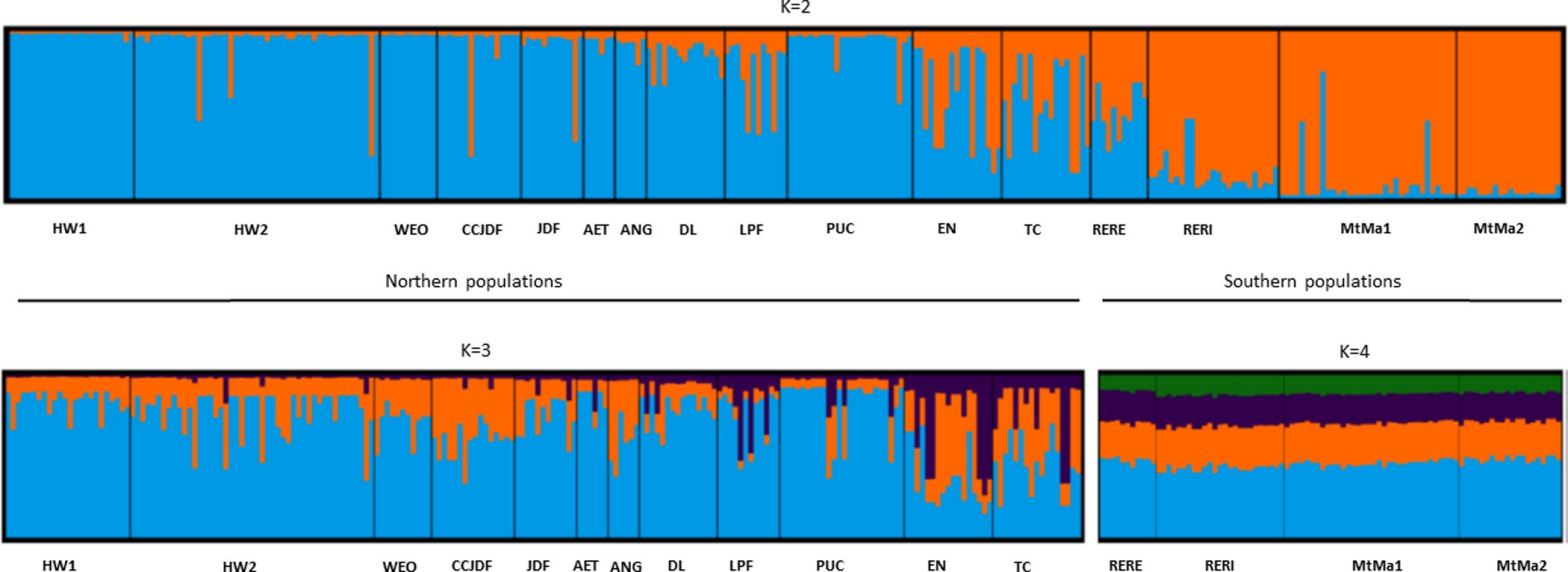
STRUCTURE analysis demonstrating the most likely number of clusters (*K*) using the “locprior” function for the French data set F based on 6 cpSSR markers.

The STRUCTURE analysis based on the six cpSSR markers suggested very low differentiation with maximum two clusters (*K* = 2) for the data sets C and F (Figs 6 and 7). The “locprior” function did not affect much the STRUCTURE results, therefore only results obtained with this function engaged are presented here. Additional STRUCTURE analysis performed separately within the northern and southern subpopulations did not find additional clusters and confirmed that the differentiation observed in the data sets F and C was mainly due to the differences between northern and southern populations. Results based on the 12 nuclear SSR markers suggested *K* = 2 and 6 with the “locprior” function engaged and disengaged for data set C, respectively, and *K* = 2 for both “locprior” function options for data set F (S7 Fig). However, the clustering was not strongly expressed (S8 Fig).

Using “assignClones” of the R-package “polysat” [43] 95 individual trees from the Burgholz stand in the data set G were identified as likely clones of the same SF72 tree from the “Sequoiafarm Kaldenkirchen”. Other individuals from Burgholz and Bad Grund were very similar to the SF76 tree, but with insufficient statistical support to assign them all unambiguously to one clone. Each clone was represented by only one individual for the diversity estimate of German samples (Table 1).

**Fig 8:**
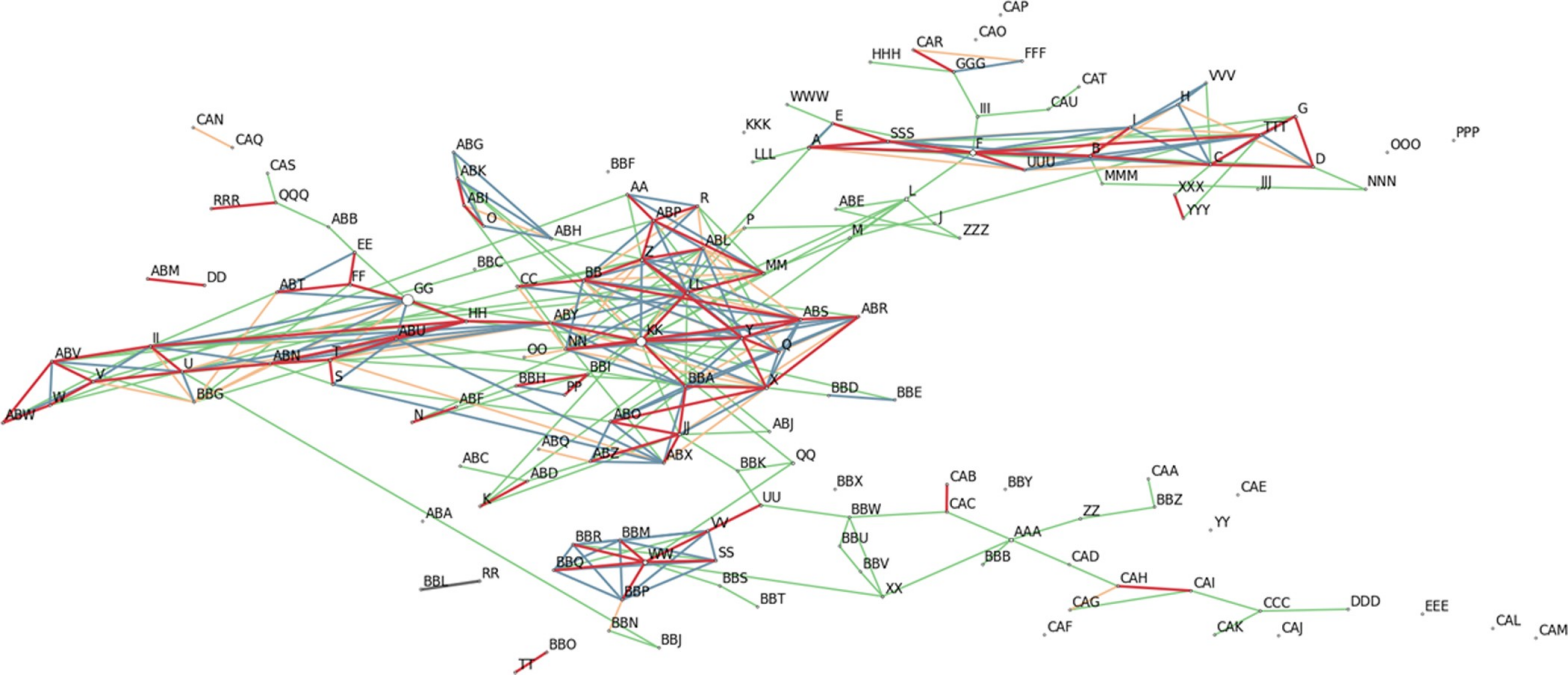
Haplotype network based on six cpSSR markers genotyped in three data sets F, C and G. Size of the nodes reflects number of individuals assigned to the respective haplotype. The percolation threshold is 2.67 Red lines present the closest links, blue to green, yellow and no colour lines indicate links following decreasing relationship between haplotypes.

Based on six cpSSR markers, 63 haplotypes were found in the data set F, 109 in C and 22 in G. In total, all three data sets together contained 150 different haplotypes (Fig 8). The haplotype network and its percolated clusters generated by the EDENetwork software indicated no geographic structure in relationships between the haplotypes (Fig 8). More than half of all haplotypes were unique—89 haplotypes occurred only once within the three data sets. The haplotypes GG and KK were the most frequent and occurred with 17% and 14% frequency, respectively.

Individuals with identical genotype representing the same clone were analysed as a single entry (S4 and S8 Tables). Accuracy of the assignments was evaluated by the quality index (QI), which was low for both F (QI = 7%) and C (QI = 16%). Most of the assignments of German trees to the southern or northern populations divided by the San Francisco Bay in the reference data set C with scores above 48% were in agreement with the assignments in the reference data set F (Tables 1 and S8). Samples with scores below 48% were assigned inconsistently between the two references, to geographically very different watersheds. For example, B11 and SF77 were assigned to RERI south of the San Francisco Bay (watershed O) based on the reference data set C, but to Del Norte close to the Oregon border (watershed B) based on reference F (Fig 1 and S8 Table). The individuals from the Sequoiafarm, which were half- or full siblings according to the owner, were assigned to various watersheds (F) and locations (C).

We used also pairwise individual tree genetic distances between all 396 trees in German (G), French (F) and Californian (C) data sets based on similarity in composition of their *Q*-values for cluster analysis to assign individual German trees to a particular watershed or a subpopulation by generating NJTs and PCoA plots, which are presented in S9–S12 Figs, respectively. The individual *Q*-values for *K* = 17 (number of watersheds) and *K* = 30 (total number of subpopulation samples or groups except German sample) and genetic distance based on them for all 396 trees in German (G), French (F) and Californian (C) data sets are presented in S9 Table. *Q*-plots based on the STRUCTURE results for *K* = 17 and *K* = 30 for all 396 trees in German (G), French (F) and Californian (C) data sets are presented in S13 Fig. The German trees closely clustered together with genetically similar trees in French and Californian data sets F and C representing different watersheds (S9 and S11 Figs) and groups (S10 and S12 Figs) are presented in Table 2.

**Table 2:** German trees in the data set G clustered together with genetically similar trees representing different watersheds (S9 and S11 Figs) and groups (S10 and S12 Figs) in French and Californian data sets F and C, respectively, based on the pairwise similarity in composition of *Q*-values obtained by STRUCTURE for *K* = 17 and *K* = 30.

*K* = 17		*K* = 30	
German trees	F and C	German trees	F and C
**SF75**	**N-G_HW_**111	**SF75**	**N-G_HW_**226
BA030549	S-N_F76	BA030549	N-G_HW_111, N-G_HW_207
**CH1**	**N-H_F136**	**CH1**	**N-H_F136**
SF76	**N**-H_F14, S-O_RERI_11	SF76	**N**-J_CCJDF_09, N-J_CCJDF_15
**GOEB**	**S-O_RERI_18**	**GOEB**	**S-O_RERI_18**
**GOEP**	**S-O_MTMA_**1B5	**GOEP**	**S**-Q_F93 | **S-O_MTMA_**208, **S**-N_RERE_01
SF3, **SF86**	**N-F_F171**, **N-F**_F174	**SF86**, SF88	**N-F_F171** | **N-F**_F172, **N**-D_F25
SF88	**N**-G_F32, **N**-K_F40		
**SF63** | **SF64** | **SF69**, **SF70** | **SF91**, **SF93**	**N-K_F129**	**SF64**, **SF93** | SF3 | **SF63**, **SF91** | **SF69**, **SF70** | SF82, SF90	**N**-I_F51 | **N-K**_F130, **N**-M_F44 | **N**-G_F29, **N-K_F129**
**SF80** | SF82, SF90	**N-F_F178**	**SF80**	**N-F_F178**
SF67, **B42**	**S-N_F52**	**B42**	**S-N_F52**
SF74	S-Q_F83	SF67, SF74	N-M_EN_15, N-M_EN_17 | N-I_F63 | N-M_AET_ANGST03, N-M_DL_D15
**SF66**, **SF77**	**N**-I_F37, **N-G_F35** | **N**-F_F167	**SF77**	**N**-C_F17, **N-G**_HW_101 | **N-G**_HW_218, **N**-M_AET_ANGST06
		**SF66**	**N**-B_F11, **N-G_F35**
**SF81** | **SF79, BG120**	**N-B**_F70 | **N-J_F38**, N-G_F160	**SF81** **SF79, BG120**	**N-B_F70,** S-Q_F83 **N-J_F38**
B30	S-O_MTMA_1A8	B30	N-G_HW_219, N-M_PUC_301
SF71	**N**-M_PUC_303	SF71	**N**-G_HW_227
SF73	**N**-M_TC_01	SF73	**N**-B_F10
B4	**N**-M_EN_08	B4	**N**-L_F57
**SF72**	**N-M**_LPF_09	**SF72**	**N-M**_EN_08
BG121	**N**-H_F137	BG121	**N**-M_DL_D04 | **N**-M_LPF_04 | **N**-M_DL_D02, **N**-M_DL_D03 | S-O_RERI_17 | **N**-G_HW_230, **N**-M_DL_D05
GOEK	N-G_HW_223		
**SF84**	**N-G_HW_245**	**SF84**	**N-G_HW_245**
**BA10067**	**S-O**_RERI_22	**BA10067**	**S-O**_MTMA_1C8
BG124 | BG123 | BG125, BG128 | B102 | BG127 | BG122, BG126	**S-O_MTMA**_1C8, **S-O_MTMA**_213 | **S-O_MTMA**_217 | **S-O_MTMA**_214	B102, BG127 | BG122 | BG126, GOEK | BG124 | BG128 | BG123, BG125	N-M_EN_13 | N-G_WEO_HRED | **S-O_MTMA**_205, **S-O_MTMA**_210

The first letter N or S in the tree ID name in the F and C data sets means North or South location, respectively. The second letter (A-Q) means watershed (see also Table 1). The trees that have F preceding the number in the name belongs to the F data set (for instance, N-H_F136 or S-Q_F83), all other trees belong to the C data (for instance, N-G_HW_111 or S-O_RERI_18). Highlighted by bold are the same area part (N or S), watershed or group in the tree names for the tress that were associated for both *K*.

## Discussion

The presented study is the first study investigating coast redwood populations within and outside the natural distribution range with a high number of nuclear SSR markers and chloroplast SSRs. The new and significantly expanded marker set confirmed results of previous studies [27,28,31]. Surprisingly, neither the novel nuclear nor chloroplast microsatellite markers could improve resolution of population differentiation within the natural distribution range of coast redwood despite high polymorphism of the used microsatellite markers. It is interesting that supposedly frost resistant German trees were assigned to both of the two clusters found within the natural range, which shows a high genetic diversity and adaptive potential in the investigated German trees.

The NJT for the data set F based on 12 nuclear SSR markers in our study was in consensus with the NJT calculated for the same 17 watersheds (A-Q) by Douhovnikoff and Dodd [31] using clones from the Russell Reserve. The discrepancies between the two trees could be explained by very low bootstrap support for most clusters in both trees and by using the pairwise *F*_ST_ values for clustering in Douhovnikoff and Dodd [31] instead of the Nei’s genetic distance used in our study. We did not use the regular pairwise *F*_ST_ for generating NJT because their values could be greatly underestimated for highly polymorphic microsatellite markers [75]. Moreover, the calculations in Douhovnikoff and Dodd were based on less than half of nuclear SSR markers (6 vs. 12), and only two were common in both studies.

The high variation within populations and low differentiation between populations are expected in wind pollinated tree species with no apparent physical or environmental barriers between populations, such as coast redwood [76]. The German samples in the data set G were closer to the northern rather than to southern populations based on both Jost’s *D*- and *F*_ST_-values (S6C Table), which makes sense, although it was not statistically significant. As expected, the level of genetic diversity and number of private alleles in GER are less than in the original populations based on nuclear SSRs (Table 1), but not as low as detected typically for recently introduced tree species [77,78].

The STRUCTURE analyses based on the cpSSR markers were able to identify the San Francisco Bay as a border or geographic barrier between two main clusters in both data sets C and F. The STRUCTURE analyses within each of the two areas, north and south of the San Francisco Bay, did not reveal any additional clusters, neither in the dataset F nor C. Both Jost’s *D*- and *F*_ST_-values are in consensus with the STRUCTURE and NJT results suggesting that CCJDF and AET in data set C and O, P and Q in data set F were the most differentiated groups compared to the remaining populations. AET represents the most interior location in data set C and surprisingly had higher genetic distance to the geographically closer populations in Napa Valley rather than to the southern populations (S6A Table).

Our data confirmed the San Francisco Bay as a border suggested already earlier by Brinegar [30] based on a single chloroplast marker. It is also identical with one of two borders identified by Sawyer et al. [79] based on the soil conditions and water availability provided by precipitation and fog. The low population structure might also be explained by transferring potentially non-local genotypes within the distribution range due to the long and intensive use of coast redwood as a timber species [80]. Trees from areas north of San Francisco Bay might have been planted in the south and vice versa. This uncertainty about the origin of genotypes and their adaptation to the local conditions should be considered in natural regeneration and conservation management strategies of coast redwood. Unfortunately, this is hard to detect and could only be verified in a comprehensive and detailed population genetic study of natural coast redwood populations in California and Oregon using genome-wide markers.

Unlike coast redwood, haplotype differentiation in other conifer species usually reflects the geographic origin of populations [81–83]. The lack of strong population structure and low genetic differentiation can explain the inconsistent assignment of German trees to populations in both reference data sets C and F and the low QI for them. The chloroplast genome is inherited paternally in coast redwood [34]. It does not prevent the cpSSRs markers to be used for the assignment, but it can be affected much by gene flow promoted by pollen and may not be as accurate as assignment based on nuclear markers. Combining the individual haplotypes closely connected together in the haplotype network into several clusters would not help for the individual assignments because individual trees composing the same cluster had different geographic origin. This conflict between geographic origin of an individual tree and position of its haplotype in the haplotype network, as well as discrepancy between data sets C and F in the assignments of German trees can be explained by the possibility that not the all sampled California stands in the data set C represented necessarily natural populations. The potential errors in the records regarding tree origin in the reference populations need to be also taken into account when considering the reliability of the assignment of individuals to an origin. Individuals with wrongly identified origin in the reference population can decrease the assignment quality [71]. However, for reliable assignment a stronger differentiation between populations in a reference data set and sufficient sample size of each reference population are needed [4,84,85]. Sample sizes were possibly insufficient for some populations in the reference C and F ranging from 4 to 47 individuals per population or watershed. However, all but five German trees were assigned correctly to the northern and southern clusters identified in the STRUCTURE analyses (Table 1, Figs 6 and 7 and S7 and S8 and S8 Table).

We also tried to find those trees in the data sets F and C which would be genetically similar to the German trees using pairwise individual similarity in their Q-value compositions to calculate pairwise genetic index of similarity and genetic distance between all trees and to visualize their clustering by generating NJTs and PCoA plots (S9–S12 Figs and S9 Table). Different German trees were similar to trees representing different watersheds (S9 and S11 Figs) and groups (S10 and S12 Figs). Some German trees were associated with the same trees or the same watershed or group in the F or C data sets for both *K* (for instance, SF75, CH1, etc.; Table 2). There is a trend of German trees to cluster more with trees from North watersheds and populations. These data can be used to trace the origin of the German trees, but cautiously. Additional verification using genome-wide markers or sequencing is needed to infer the true origin of the German trees.

The differentiation between populations based on the cpSSR markers is usually less than based on nuclear SSRs in conifers [86]; particularly, due to long distance migration by pollen and because the mutation rate in chloroplast microsatellite loci is lower [87]. In case of coast redwood, the paternal inheritance of chloroplasts [34] and the long distance gene flow via wind-dispersed pollen and seeds could explain the similarly low differentiation results for both marker sets. Ribeiro et al. [88] found similar results when comparing population differentiation based on AFLP markers with the one based on the paternally inherited cpSSR markers in the wind pollinated conifer *Pinus pinaster* Aiton. In addition, Petit et al. [89] showed that for various conifer species genetic differentiation based on bi-parentally inherited markers correlated with differentiation based on paternally inherited markers due to the similar gene flow vectors, but the latter was lower in general [90].

The reliable clone identification based on microsatellites in data set G (S6 Fig) confirmed results of previous studies based on allozyme and AFLP markers [27,91]. General difficulties to accurately genotype microsatellite markers in polyploid organisms excludes analyses based on their allele frequencies [35,92–95]. It concerns also coast redwood, but genotyping problems could be even more aggravated due to a high probability of somatic mutations in basal sprouting shoots in these extremely long living trees, which can result in different genotypes of different tissues and clones originated from the same tree [35]. The somatic mutations can help to maintain a high genetic diversity level in coast redwood populations, despite the low seed germination rate and high clonal growth. The assumption of Hardy-Weinberg equilibrium is also tricky due to common clonal growth in coast redwood populations [92], where trees within 40 m radius can belong to the same clone [91].

The correct estimation of the null allele frequencies and allelic dosage are two major difficulties associated with genotyping polyploid organisms using microsatellite markers [32,37]. In our study, the risk of null alleles should be reduced since null alleles are in general less frequent in EST-SSRs due to their location in more conserved regions [32], and the Bruvo distance used as the genetic distance measure in this study does not require same allelic dosages between individuals [43,45]. The fragment scoring following Pfeiffer et al. [42] and confirmation of allele scoring using multiple ramets of the same clones reduced the scoring errors due to the stutter effects in this study. Narayan et al. [35] considered only those alleles for the multilocus lineages (MLL) that were consistent in at least two different tissue types of the same tree, and they found a low error rate in allelic dosage in coast redwood based on the Bruvo distance. The possible number of genotypes in polyploid species, such as coast redwood, can be very high [42], hence, just a few microsatellite markers can be sufficient for clonal identification and population structure analysis.

The number of chloroplast haplotypes found in this study was exceptionally high (150) based on six cpSSR markers genotyped in 579 samples in total in all data sets. This extremely high number of chloroplast haplotypes was not observed earlier in any tree species. Similar high genetic diversity in chloroplasts was observed in another conifer species, *Abies nordmanniana* (Steven) Spach., with 111 haplotypes in 361 individuals, although the sampling range included a several times larger area than for coast redwood [96]. Although speculative, there could be several reasonable explanations such as plastid heteroplasmy and accumulation of somatic mutations during the long lifespan of coast redwood, which need additional research for verification. Plastid heteroplasmy has been observed in several plant species and can occur also due to bi-parental inheritance, hybridization, introgression, recombination between chloroplast genomes and plastid leakage [97,98]. According to Wolfe and Randle [99], heteroplasmy due to hybridization can be a stable condition. Additionally, long-living perennials and vegetatively reproducing plants can maintain a high genetic variation level in chloroplasts [99]. Scott et al. [100] did not rule out an allopolyploidization event within the “Sequoia-clade” as a source of the polyploidy of coast redwood. Possible hybridization events, longevity of trees and clonal propagation as a source and maintenance of chloroplast heteroplasmy apply to coast redwood. One can expect multiple fragments representing different chloroplast alleles amplified due to chloroplast heteroplasmy, but we observed exclusively single fragments in this study. However, it might be due to the uneven allelic dosage within a sample, and as a result only one allele (fragment) is amplified during PCR [99]. To our knowledge, we are unaware of any studies specifically demonstrating correlation between polyploidy and chloroplast heteroplasmy in plants; although, in the genus *Medicago* both polyploidy and chloroplast heteroplasmy are common [101]. However, finding the reason for the exceptionally high number of haplotypes in coast redwood requires further investigations.

## Conclusions and future directions

Coast redwood forests used to have a continuous distribution along the Pacific Coast in California before intensive logging started at the beginning of the nineteenth century [102]. Therefore, current coast redwood populations are considered as remnant and fragmented populations. However, being long living clonal trees with a high somatic mutation rate, coast redwood maintained its high genetic diversity despite multiple bottlenecks [15,34]. Although a limited number of German trees were introduced only decades ago, they have relatively high genetic diversity.

The combination of low sexual reproduction and local adaptation could cause insufficient ability to meet the challenges of climate change and will increase the pressure on coast redwood [9]. California has become drier in the last 2000 years [16], and the very important fog has been declining in its frequency during the last century [39,103]. Considering these threats O’Hara et al. [15] emphasized the necessity to find drought tolerant genotypes and to increase genetic diversity, especially for southern populations. Genetic and physiological mechanisms behind drought resistance are similar to those that are behind frost tolerance, therefore geographic variation in drought tolerance in tree species often overlaps with variation in frost tolerance [104–106]. The identification of water stress resistant genotypes would benefit both Californian and German forestry. Tolerant genotypes would not only provide Germany with a valuable timber species considering climate change, but also presents suitable resources for *ex-situ* conservation programs for coast redwood, which was already suggested for the sister species *Sequoiadendron giganteum* (Lindl.) J. Buchholz [8].

Further studies of the genetic structure of coast redwood populations based on functional markers that are potentially under selection, such as non-synonymous SNPs or SNPs in regulatory genes, can help to identify local adaptation to particular environmental stress factors of interest [107]. More studies should be also done using mitochondrial DNA markers, which can help to infer maternally based gene flow and lineages due to their maternal inheritance. They are distributed by seeds and should be less affected by long-distance dispersal than pollen [108]. The results obtained in these prospective studies will provide additional important data for sustainable timber production and conservation management, *in situ* and *ex situ*, for coast redwood, especially in the context of predicted climate change.

## Supporting information

S1 Fig Map of the original “Kuser’s” samples in the data set F. Original locations of the “Kuser’s” samples in the data set F are presented on the map together with the mean monthly temperature pattern for the time period 1979–2013 indicating colder and warmer temperatures by darker and lighter shades of grey (http://chelsa-climate.org).
(PDF)

S2 Fig Map of the German sampling locations (data set G). Darker and lighter shades of grey highlight colder and warmer mean monthly temperatures for the time period 1979–2013 (http://chelsa-climate.org).
(PDF)

S3 Fig Neighbour-joining tree of 84 ramets representing 30 different clones (with 2–3 ramets per clone) provided by the Allerweltsgrün nursery (Köln). It is based on 12 nSSR markers with the final ranking combination obtained according to Pfeiffer et al. [45] and the ‘bruvo genetic distance’ [48]. Numbers indicate bootstrap values (not percentage). The sample ID represents the clone ID followed by the ramet consecutive number.
(PDF)

S4 Fig Neighbour-joining tree of the “Kuser’s” samples in the French (F) data set (St. Fargeau) grouped into 17 watersheds (A-Q). It is based on six cpSSR markers and Nei’s genetic distance ([50] after [51]) with 1000 bootstraps. Numbers indicate bootstrap values in terms of percentage.
(PDF)

S5 Fig Neighbour-joining tree of the 16 Californian reference populations represented by samples collected in 2017 (data set C). It is based on six cpSSR markers and Nei’s genetic distance ([50] after [51]) with 1000 bootstraps. Numbers indicate bootstrap values in terms of percentage. County names of sampling sites are in brackets.
(PDF)

S6 Fig Neighbour-joining tree of the 143 individual trees collected in Germany (data set G). It is based on 12 nSSR markers with the final ranking combination obtained according to Pfeiffer et al. [45] and the ‘bruvo genetic distance’ [48] with 1000 bootstraps. Numbers indicate bootstrap values (not percentage).
(PDF)

S7 Fig STRUCTURE results for the reference data sets C and F based on 12 nSSR markers. Plots for data sets C and F are presented for the most likely number of clusters (*K*) inferred without (a) and with (b) the “locprior” function engaged (*K* = 6 and *K* = 2 for C and *K* = 2 for F, respectively). L(*K*) and Δ*K* statistics generated by the ClumPAK software are also presented.
(PDF)

S8 Fig STRUCTURE statistics for the reference data sets C and F based on 6 cpSSR markers. Presented are the most likely number of clusters (*K*) and results of STRUCTURE runs for *K* from 1 to 20 or 25 with 20 iterations each for the complete data set C (a), northern subset C (b), southern subset C (c), complete data set F (d), northern subset F (e), and southern subset F (f).
(PDF)

S9 Fig Neighbour-joining trees (NJTs) based on pairwise individual tree genetic distances between all 396 trees in German (G), French (F) and Californian (C) data sets generated using *Q*-values for *K* = 17. (PDF)

S10 Fig Neighbour-joining trees (NJTs) based on pairwise individual tree genetic distances between all 396 trees in German (G), French (F) and Californian (C) data sets generated using *Q*-values for *K* = 30. (PDF)

S11 Fig Principal coordinate analysis (PCoA) plots based on pairwise individual tree genetic distances between all 396 trees in German (G), French (F) and Californian (C) data sets generated using Q-values for K = 17. (PDF)

S12 Fig Principal coordinate analysis (PCoA) plots based on pairwise individual tree genetic distances between all 396 trees in German (G), French (F) and Californian (C) data sets generated using *Q*-values for *K* = 30. (PDF)

S13 Fig *Q*-plots based on the STRUCTURE results for *K* = 17 and *K* = 30 for all 396 trees in German (G), French (F) and Californian (C) data sets. (PDF)

S1 Table ID, geographic and watershed data for the French (F) set of the “Kuser’s” samples. (DOCX)

S2 Table Original latitude and number of samples representing 17 watersheds in the French (F) data set (St. Fargeau) and the Russell Reserve according to Douhovnikoff and Dodd [34]. (DOCX)

S3 Table Geographic and climatic data for sampled locations in California (data set C). (DOCX)

S4 Table Sampling site, clone ID, and geographic location for samples collected in Germany (data set G). (XLSX)

S5 Table Data for 18 microsatellite (SSR) markers used in the study. (DOCX)

S6 Table Pairwise Jost's D- (in the left corner lower triangle) and FST- (in the right upper corner triangle) values based on 12 nSSR marker for 16 populations of data set C (A), 17 watersheds of data set F (B), and among three groups NORTH, SOUTH, and GER, respectively (C). All values were insignificant.
(XLSX)

S7 Table Verification of the 18 PCR primer pairs of the 18 microsatellite (SSR) markers by mapping their primer nucleotide sequences to the draft coast redwood nuclear and chloroplast genome assemblies. (XLSX)

S8 Table The German samples in the data set G assigned to the populations and watersheds in the reference data sets representing samples from California (C) and the “Kuser Provenance test” in St. Fargeau, central France (F), respectively, and assignment quality scores (%) for two ranks. (DOCX)

S9 Table *Q*-matrices for *K* = 17 and *K* = 30 and genetic distance matrices based on them. (XLSX)

## References

[1] Lindner M, Maroschek M, Netherer S, Kremer A, Barbati A, et al Climate change and European forests: What do we know, what are the uncertainties, and what are the implications for forest management? For Ecol Manage. 2010; 259: 698–709.

[2] Pompe S, Hanspach J, Badeck F, Klotz S, Thuiller W, Kühn I. Climate and land use change impacts on plant distributions in Germany. Biol Lett. 2008; 4(5): 519–531. 10.1098/rsbl.2008.0231 18664416 PMC2610074

[3] Neuner S T. Knoke T. Economic consequences of altered survival of mixed or pure Norway spruce under a dryer and warmer climate. Clim Change. 2017; 140(3–4): 519–531.

[4] Hintsteiner WJ, van Loo M, Neophytou C, Schueler S, Hasenauer H. The geographic origin of old Douglas-fir stands growing in Central Europe. Eur J For Res. 2018; 137(4): 447–461.

[5] Chakraborty D, Wang T, Andre K, Konnert M, et al Adapting Douglas-fir forestry in Central Europe: evaluation, application, and uncertainty analysis of a genetically based model. Eur J For Res. 2016; 135(5): 919–936.

[6] Urli M, Delzon S, Eyermann A, Couallier V, et al Inferring shifts in tree species distribution using asymmetric distribution curves: A case study in the Iberian mountains. J Veg Sci. 2014; 25(1): 147–159.

[7] Ahuja MR. Woody Plant Biotechnology. Plenum Press, New York and London 1991.

[8] Ahuja MR. Genetic constitution and diversity in four narrow endemic redwoods from the family Cupressaceae. In: Biodiversity and Conservation of woody plants. Springer International Publishing. 2017; 165(1): 5–19.

[9] Ahuja MR. Strategies for conservation of germplasm in endemic redwoods in the face of climate change: A review. Plant Genet Resour-C. 2011; 9(3): 411–422.

[10] Roy DF. Silvical characteristics of redwood (Sequoia sempervirens Endl.). U.S. Forest Service Research Paper. 1966; PSW 28:20.

[11] Busing RT, Fujimori T. Biomass, production and woody detritus in an old coast redwood (*Sequoia sempervirens*) forest. Plant Ecol. 2005; 177(2): 177–188.

[12] Jones DA, O’Hara KL. Carbon density in managed coast redwood stands: Implications for forest carbon estimation. Forestry. 2012; 85(1): 99–110.

[13] Arnaud Y, Franclet A, Tranvan H, Jacques M. Micropropagation and rejuvenation *Sequoia sempervirens* (Lamb) Endl: a review. Ann For Sci. 1993; 50(8):273–295.

[14] Palmer DJ, Watt MS, Kimberley MO, Dungey HS. The spatial distribution of Sequoia sempervirens productivity in New Zealand. New Zealand J For Sci. 2012; 42: 81–89.

[15] O’Hara KL, Cox LE, Nikolaeva S, Bauer JJ, Hedges R. Regeneration dynamics of coast redwood, a sprouting conifer species: A review with implications for management and restoration. Forests. 2017; 8(5): 144.

[16] Lorimer CG, Porter DJ, Madej MA, Stuart JD, Veirs SD Jr., et al Presettlement and modern disturbance regimes in coast redwood forests: Implications for the conservation of old-growth stands. For Ecol Manage. 2009; 258(7): 1038–1054.

[17] Dawson TE. Redwood forest: Fog in the California ecosystem inputs and use by plants. Oecologia. 1998; 117(4):476–485. 10.1007/s004420050683 28307672

[18] Templer PH, Weathers K, Ewing H, Dawson HA, Mambelli TE et al Fog as a source of nitrogen for redwood trees: Evidence from fluxes and stable isotopes. J Ecol. 2015; 3(6):1397–1407.

[19] U.S. Department of Agruiculture (USDA). 2012. Available from: https://planthardiness.ars.usda.gov/phzmweb/interactivemap.aspx [Cited 28 June 2019].

[20] Pettenkofer T, Burkardt K, Ammer C, Vor T, Finkeldey R, Müller M, et al Genetic diversity and differentiation of introduced red oak (*Quercus rubra*) in Germany in comparison with reference native North American populations. Eur J For Res. 2019; 138: 275–285.

[21] Verhaegen D, Fofana IJ, Zénor AL, Ofori D. What is the genetic origin of teak (*Tectona grandis* L.) introduced in Africa and in Indonesia? Tree Genet Genomes. 2010; 6: 717–733.

[22] Nazareno AG, Dos Reis MS. Where did they come from? Genetic diversity and forensic investigation of threatened palm species *Butia eriospatha*. Conserv Genet. 2014; 15: 441–452.

[23] Rogers DL. Inheritance of allozymes from seed tissues of the hexaploid gymnosperm, *Sequoia sempervirens* (D. Don) Endl. (coast redwood). Heredity. 1997; 78: 166–175.

[24] Rogers DL. Genotypic diversity and clone size in old-growth populations of coast redwood (*Sequoia sempervirens*). Can J Bot. 2000; 78: 1408–1419.

[25] Rogers DL, Westfall RD. Spatial genetic patterns in four old-growth populations of coast redwood. In: Standiford, Richard B, Giusti, Gregory A, Valachovic Y, Zielinski WJ, Furniss MJ. technical Editors. Proceedings of the redwood region forest science symposium: What does the future hold? Gen. Tech. Rep. PSW-GTR-194. Albany, CA: Pacific Southwest Research Station, Forest Service, U.S. Department of Agriculture. 2007; 59–64.

[26] Bruno D, Brinegar C. Microsatellite markers in coast redwood (*Sequoia sempervirens*). Mol Ecol Notes. 2004; 4: 482–484.

[27] Toral Ibañez M, Manníquez A, Navarro-Cerrillo R, Tersi D, Naulin P, et al Clones identification of *Sequoia sempervirens* (D. Don) Endl. in Chile by using PCR-RAPDs technique. J Zhejiang Univ Sci B. 2009; 10(2): 112–119. 10.1631/jzus.B0820162 19235269 PMC2644751

[28] Rogers DL. Allozyme polymorphisms discriminate among coast redwood (*Sequoia sempervirens*) siblings. J Hered. 1999; 90(3): 429–433.

[29] Douhovnikoff V, Cheng AM, Dodd RS. Size and spatial structure of clones in second—growth stands of coast redwood. Nucleic Acids Res. 2004; 91(7): 1140–1146. 10.3732/ajb.91.7.1140 21653469

[30] Brinegar C. Rangewide genetic variation in coast redwood populations at a chloroplast microsatellite locus. In: Standiford RB, Weller TJ, Piierto DD, Stuart JD, editors. Proceedings of the Coast Redwood Forests in A Changing California: A Symposium for Scientists and Managers. USDA Forest Service General Technical Report PSW-GTR-238. Albany, California, USA: USDA Forest Service, Pacific Southwest Research Station. 2011; pp. 241–249.

[31] Douhovnikoff V, Dodd RS. Lineage divergence in coast redwood (*Sequoia sempervirens*), detected by a new set of nuclear microsatellite loci. Am Midl Nat. 2011; 165: 22–37.

[32] Ellis JR, Burke JM. EST-SSRs as a resource for population genetic analyses. Heredity. 2007; 99(2): 125–132. 10.1038/sj.hdy.6801001 17519965

[33] Provan J, Powell W, Hollingsworth PM. Chloroplast microsatellites: new tools for studies in plant ecology and evolution. Trends Ecol Evol. 2001; 16(3): 142–147. 10.1016/s0169-5347(00)02097-8 11179578

[34] Neale DB, Marshall KA, Sederoff RR. Chloroplast and mitochondrial DNA are paternally inherited in *Sequoia sempervirens* D. Don Endl. Proc Natl Acad Sci USA. 1989; 86(23): 9347–9349. 10.1073/pnas.86.23.9347 16594091 PMC298492

[35] Narayan L, Dodd RS, O’Hara KL. A genotyping protocol for multiple tissue types from the polyploid tree species *Sequoia sempervirens* (Cupressaceae). Appl Plant Sci. 2015; 3: 1–7. 10.3732/apps.1400110 PMC4356318 25798341

[36] Breidenbach N, Gailing O, Krutovsky KV. Development of novel polymorphic nuclear and chloroplast microsatellite markers in coast redwood (*Sequoia sempervirens*). Plant Genet Resour-C. 2019; 17(3): 293–297. 10.1017/S147926211800045X.

[37] Kuser JE, Bailly A, Franclet A, Libby WJ, Martin J, et al Early results of a rangewide provenance test of *Sequoia sempervirens*. For Genet Resour. 1995; 23: 1–5.

[38] Olson DF Jr, Roy DF, Walters GA. Sequoia sempervirens (D.Don) Endl. Redwood In: Burns RM, Honkala BH, Eds. Technical coordinators. Silvics of North America, Volume 1, conifers, Agriculture handbook 654. Washington, DC, USA USDA Forest Service 1990; 541–551.

[39] Torregrosa A, Combs C, Peters J. GOES-derived fog and low cloud indices for coastal north and central California ecological analyses. Earth Space Sci. 2015; 3(2): 46–67. 10.1002/2015EA000119.

[40] Stebbins GLJ. The Chromosomes and Relationships of *Metasequoia* and *Sequoia*. Science. 1948; 108: 95–98. 10.1126/science.108.2796.95 17808724

[41] Mason AS. Challenges of genotyping polyploid species In: Batley J, ed. Plant Genotyping Methods and Protocols. Humana Press Springer Science+Busines Media New York 2015; 161–168. 10.1007/978-1-4939-1966-6_12 25373756

[42] Pfeiffer TA, Roschanski AM, Pannell JR, Korbecka G, Schnittler M. Characterization of microsatellite loci and reliable genotyping in a polyploid plant, *Mercurialis perennis* (Euphorbiaceae). J Hered. 2011; 102(4): 479–488. 10.1093/jhered/esr024 21576288

[43] Clark LV, Jasieniuk M. Polysat: An R package for polyploid microsatellite analysis. Mol Ecol Resour. 2011; 11(3): 562–566. 10.1111/j.1755-0998.2011.02985.x 21481215

[44] R Core Team. R: A language and environment for statistical computing. R Foundation for Statistical Computing, Vienna, Austria 2018 https://www.R-project.org.

[45] Bruvo R, Michiels NK, D’Souza TG, Schulenburg H. A simple method for the calculation of microsatellite genotype distances irrespective of ploidy level. Mol Ecol. 2004; 13(7): 2101–2106. 10.1111/j.1365-294X.2004.02209.x 15189230

[46] Paradis E, Schliep K. ape 5.0: an enivronment for modern phylogenetics and evolutionary analyses in R. Bioinformatics. 2019; 35(3): 526–528. 10.1093/bioinformatics/bty633 30016406

[47] Nei M. Genetic distance between populations. Am Nat. 1972; 106: 283–291.

[48] Lynch M, Milligan BG. Analysis of population genetic structure with RAPD markers. Mol Ecol. 1994; 3: 91–99. 10.1111/j.1365-294x.1994.tb00109.x 8019690

[49] Vekemans X. AFLP-SURV version 1.0: a program for genetic diversity analysis with AFLP (and RAPD) population data. Distributed by the author. Laboratoire de Génétique et Ecologie Végétale, Université Libre de Bruxelles, Belgium 2002; http://www.ulb.ac.be/sciences/lagev/aflp-surv.html.

[50] Felsenstein J. PHYLIP (Phylogeny Inference Package) version 3.6. Distributed by the author. Department of Genome Sciences, University of Washington, Seattle.

[51] Rambaut A. FigTree v1.3.1. Institute of Evolutionary Biology, University of Edinburgh, Edinburgh http://tree.bio.ed.ac.uk/software/figtree.

[52] Jombart T. adegenet: An R package for the multivariate analysis of genetic markers. Bioinformatics. 2008; 24: 1403–1405. 10.1093/bioinformatics/btn129 18397895

[53] Jombart T, Ahmed I. adegenet 1.3–1: new tools for the analysis of genome-wide SNP data. Bioinformatics.2011; 27(21):3070–3071. 10.1093/bioinformatics/btr521 21926124 PMC3198581

[54] Kamvar ZN, Tabima JF, Grünwald NJ. Poppr: An R package for genetic analysis of populations with clonal, partially clonal and/or sexual reproduction. PeerJ. 2014; 2: e281 10.7717/peerj.281 24688859 PMC3961149

[55] Kamvar ZN, Brooks JC, Grünwald NJ. Novel R tools for analysis of genome-wide population genetic data with emphasis on clonality. Front Genet. 2015; 6: 208 10.3389/fgene.2015.00208 26113860 PMC4462096

[56] Peakall R, Smouse PE. GenAlEx 6.5: genetic analysis in Excel. Population genetic software for teaching and research-an update. Bioinform. 2012; 28: 2537–2539. 10.1093/bioinformatics/bts460 22820204 PMC3463245

[57] Pritchard JK, Stephens M, Donnelly P. Inference of population structure using multilocus genotype data. Genetics. 2000; 76: 170–181. 10835412 10.1093/genetics/155.2.945 PMC1461096

[58] Falush D, Stephens M, Pritchard JK. Inference of population structure: Extensions to linked loci and correlated allele frequencies. Genetics. 2003; 164: 1567–1587. 12930761 10.1093/genetics/164.4.1567 PMC1462648

[59] Falush D, Stephens M, Pritchard JK. Inference of population structure using multilocus genotype data: dominant markers and null alleles. Mol Ecol Notes. 2007; 7: 574–578. 10.1111/j.1471-8286.2007.01758.x 18784791 PMC1974779

[60] Hubisz M, Falush D, Stephens M, Pritchard J. Inferring weak population structure with the assistance of sample group information. Mol Ecol Resour. 2009; 9: 1322–1332. 10.1111/j.1755-0998.2009.02591.x 21564903 PMC3518025

[61] Evanno G, Regnaut S, Goudet J. Detecting the number of clusters of individuals using the software STRUCTURE: a simulation study. Mol Ecol. 2005; 14: 2611–2620. 10.1111/j.1365-294X.2005.02553.x 15969739

[62] Earl DA, von Holdt BM. STRUCTURE HARVESTER: a website and program for visualizing STRUCTURE output and implementing the Evanno method. Cons Genet Resour. 2012; 4: 359–361. 10.1007/s12686-011-9548-7.

[63] Kopelman NM, Mayzel J, Jakobsson M, Rosenberg NA, Mayrose I. CLUMPAK: a program for identifying clustering modes and packaging population structure inferences across K. Mol Ecol Resour. 2015; 5: 1179–1191. 10.1111/1755-0998.12387 25684545 PMC4534335

[64] Zhivotovsky LA. Index of population similarity in polymorphic traits. Zhurnal Obshchei Biologii. 1979; 40(4): 587–602.

[65] Goldstein DB, Linares AR, Cavalli-Sforza LL, Feldman MW. Genetic absolute dating based on microsatellites and the origin of modern humans. Proc Natl Acad Sci USA. 1995; 92: 6723–6727. 10.1073/pnas.92.15.6723 7624310 PMC41401

[66] Kivelä M, Arnaud-Haond S, and J. Saramäki J. EDENetworks: A user-friendly software to build and analyse networks in biogeography, ecology and population genetics. Mol Ecol Resour. 2015; 15(1): 117–122. 10.1111/1755-0998.12290 24902875

[67] Stauffer D, Aharony A. Introduction to Percolation Theory. 1994 London.

[68] Piry S, Alapetite A, Cornuet JM, Paetkau D, Baudouin L, Estoup A. GENECLASS2: A software for genetic assignment and first-generation migrant detection. J Hered. 2004; 95(6): 536–539. 10.1093/jhered/esh074 15475402

[69] Rannala B, Mountain JL. Detecting immigration by using multilocus genotypes. Proc Natl Acad Sci USA. 1997; 94: 9197–9201. 10.1073/pnas.94.17.9197 9256459 PMC23111

[70] Paetkau D, Slade R, Burden M, Estoup A. Direct, real-time estimation of migration rate using assignment methods: a simulation-based exploration of accuracy and power. Mol Ecol. 2004; 13: 55–65. 10.1046/j.1365-294x.2004.02008.x 14653788

[71] Cornuet JM, Piry S, Luikart G, Estoup A, Solignac M. New methods employing multilocus genotypes to select or exclude populations as origins of individuals. Genetics. 1999; 153: 1989–2000. 10581301 10.1093/genetics/153.4.1989 PMC1460843

[72] Blanchard JL, Schmidt GW. Pervasive migration of organellar DNA to the nucleus in plants. J Mol Evol. 1995; 41: 397–406. 10.1007/BF00160310 7563126

[73] Takanori Y, Hazuka Y, Furihata, Kawabe A. Patterns of genomic integration of nuclear chloroplast DNA fragments in plant species. DNA Research. 2014; 21(2): 127–140. 10.1093/dnares/dst045 24170805 PMC3989485

[74] Rousseau-Gueutin M, Keller J, Ferreira de Carvalho J, Aϊnouche A, Martin G. The interwined chloroplast and nuclear genome coevolution in plants in: Ratnadewi D, Hamim (Eds). Plants Growth and Regulation- alterations to sustain unfavorable conditions. 2018 INTECHOPEN LIMITED, London, United Kingdom.

[75] Meirmans PG, Hedrick PW. Assessing population structure: *F*_ST_ and related measures. Mol Ecol Resour. 2011; 11: 5–18. 10.1111/j.1755-0998.2010.02927.x 21429096

[76] Krutovsky KV, Burczyk J, Chybicki I, Finkeldey R, Pyhäjärvi T, Robledo-Arnuncio JJ. Gene flow, spatial structure, local adaptation, and assisted migration in trees In: Schnell R, Priyadarshan P, editors. Genomics of Tree Crops. Springer, New York, NY; 2012 pp. 71–116. 10.1016/j.jenvrad.2011.11.013

[77] Nei M, Maruyama T, Chakraborty R. The bottleneck effect and genetic variability in populations. Evolution.1975; 29: 1–10. 10.1111/j.1558-5646.1975.tb00807.x 28563291

[78] Barrett SC, Husband BC. The genetics of plant migration and colonization In: Brown HD, Clegg MT, Kahler AL et al, editors. Plant population genetics, breeding, and genetic resources. Sinauer Associates Inc, Massachusetts; 1990 pp 254–277.

[79] Sawyer JO, Sillett SC, Popenoe JH, LaBanca A, Sholars T, Largent DL, et al Characteristics of Redwood Forests in Noss RF, editors. The Redwood Forest. Island Press, Washington DC, USA; 2000.

[80] Stephens SL, Fry DL. Fire History in Coast Redwood Stands in the Northeastern Santa Cruz Mountains, California. Fire Ecology. 2005; 1(1): 18.

[81] Echt CS, DeVerno LL, Anzidei M, Vendramin GG. Chloroplast microsatellites reveal population genetic diversity in red pine, *Pinus resinosa* Ait. Mol Ecol. 1998; 7: 307–316.

[82] Vendramin GG, Anzidei M, Madaghiele A, Sperisen C, Bucci G. Chloroplast microsatellite analysis reveals the presence of population subdivision in Norway spruce (*Picea abies* K.). Genome. 2000; 43: 68–78. 10701115

[83] Terrab A, Talavera S, Arista M, Paun O, Stuessy TF, Tremetsberger K. Genetic diversity at chloroplast microsatellites (cpSSRs) and geographic structure in endangered West Mediterranean firs (Abies spp., Pinaceae). Taxon 2007; 56 (2): 409–416.

[84] Ribeiro MM, LeProvost G. Gerber S, Vendramin GG, et al Origin identification of maritime pine stands in France using chloroplast simple-sequence repeats. Ann For Sci. 2002; 59: 53–62.

[85] Bjørnstad G, Røed KH. Evaluation of factors affecting individual assignment precision using microsatellite data from horse breeds and simulated breed crosses. Anim Genet. 2002; 33 (4): 264–270. 10.1046/j.1365-2052.2002.00868.x 12139505

[86] Petit RJ, Deguilloux MF, Chat J, Grivet D, Garnier-Géré P, Vendramin GG. Standardizing for microsatellite length in comparisons of genetic diversity. Mol Ecol. 2005; 14(3): 885–890. 10.1111/j.1365-294X.2005.02446.x 15723680

[87] Provan J, Soranzo N, Wilson NJ, Goldstein DB, Powell W. A low mutation rate for chloroplast microsatellites. Genetics. 1999; 153(2): 943–947. 10511569 10.1093/genetics/153.2.943 PMC1460781

[88] Ribeiro MM, Mariette S, Vendramin GG, Szmidt AE, Plomion C, Kremer A. Comparison of genetic diversity estimates within and among populations of maritime pine using chloroplast simple-sequence repeat and amplified fragment length polymorphism data. Mol Ecol. 2002; 11(5): 869–877. 10.1046/j.1365-294x.2002.01490.x 11975703

[89] Petit RJ, Duminil J, Fineschi S, Hampe A, Salvini D, Vendramin GG. Comparative organization of chloroplast, mitochondrial and nuclear diversity in plant populations. Mol Ecol. 2005; 14: 689–701. 10.1111/j.1365-294X.2004.02410.x 15723661

[90] Provan J, Beatty GE, Hunter AM, McDonald RA, et al Restricted gene flow in fragmented populations of a wind-pollinated tree. Conser Genet. 2008; 9(6): 1521–1532.

[91] Douhovnikoff V, Cheng AM, Dodd RS. Incidence, size and spatial structure of clones in second-growth stands of coast redwood, Sequoia sempervirens (Cupressaceae). Am J Bot. 2004; 91(7): 1140–1146. 10.3732/ajb.91.7.1140 21653469

[92] Dufresne F, Stift M, Vergilino R, Mable BK. Recent progress and challenges in population genetics of polyploid organisms: An overview of current state-of-the-art molecular and statistical tools. Mol Ecol. 2014; 23(1): 40–69. 10.1111/mec.12581 24188632

[93] Nybom H. Comparison of different nuclear DNA markers for estimating intraspecific genetic diversity in plants. Mol Ecol. 2004; 13(5): 1143–1155. 10.1111/j.1365-294X.2004.02141.x 15078452

[94] Esselink GD, Nybom H, Vosman B. Assignment of allelic configuration in polyploids using the MAC-PR (microsatellite DNA allele counting—Peak ratios) method. Theor Appl Genet. 2004; 109(2): 402–408. 10.1007/s00122-004-1645-5 15085263

[95] De Silva HN, Hall AJ, Rikkerink E, McNeilage MA, Fraser LG. Estimation of allele frequencies in polyploids under certain patterns of inheritance. Heredity. 2005; 95(4): 327–334. 10.1038/sj.hdy.6800728 16094298

[96] Hansen OK, Kjær ED, Vendramin GG. Chloroplast microsatellite variation *in Abies nordmanniana* and simulation of causes for low differentiation among populations. Tree Genet Genomes. 2005;1(3): 116–123.

[97] Harris SA, Ingram R. Chloroplast DNA and Biosystematics: the effects of intraspecific diversity and plastid transmission. Taxon. 1991; 40(3): 393–412.

[98] Lambertini C. Heteroplasmy due to chloroplast paternal leakage: another insight into *Phragmites* haplotypic diversity in North America. Biological Invasions. 2016; 18: 2443–2455. 10.1007/s10530-016-1193-3.

[99] Wolfe AD, Randle CP. Recombination, heteroplasmy, haplotype polymorphism, and paralogy in plastid genes: Implications for plant molecular systematics. Syst Bot. 2004; 29(4): 1011–1020.

[100] Scott AD, Stenz NWM, Ingvarsson PK, Baum DA. Whole genome duplication in coast redwood (Sequoia sempervirens) and its implications for explaining the rarity of polyploidy in conifers. New Phytol. 2016; 211(1): 186–193. 10.1111/nph.13930 26996245

[101] Johnson LB, Palmer JD. Heteroplasmy of chloroplast DNA in *Medicago*. Plant Mol Biol. 1989; 12: 3–11. 10.1007/BF00017442 24272712

[102] Sawyer JO, Gray JG, West GJ, Thornburgh DA, Noss RF, Engbeck JH, et al History of redwood and redwood forests In: Noss RF, editors. The Redwood Forest. Island Press, Washington DC, USA; 2000.

[103] Johnstone JA, Dawson TE. Climatic context and ecological implications of summer fog decline in the coast redwood region. Proceedings National Academy of Science.2010; 107(10): 4533–4538. 10.1073/pnas.0915062107 20160112 PMC2822705

[104] Beck EH, Fettig S, Knake C, Hartig K, Bhattarai T. Specific and unspecific responses of plants to cold and drought stress. J Biosci. 2007; 32: 501–510. 10.1007/s12038-007-0049-5 17536169

[105] Howe GT, Aitken SN, Neale DB, Jermstad KD, Wheeler NC, Chen TH. From genotype to phenotype: unraveling the complexities of cold adaptation in forest trees. Can J Bot. 2003; 81(12): 1247–1266.

[106] Sakai A, Larcher W. Frost survival of plants. 1987; 62 Springer Verlag Berlin Heidelberg.

[107] Sork VL, Aitken SN, Dyer RJ, Eckert AJ, Legendre P, Neale DB. Putting the landscape into the genomics of trees: approaches for understanding local adaptation and population responses to changing climate. Tree Genet Genomes. 2013; 9: 901–911.

[108] Potter KM, Hipkins VD, Mahalovich MF, Means RE. Mitochondrial DNA haplotype distribution patterns in Pinus ponderosa (Pinaceae): Range-wide evolutionary history and implications for conservation. Am J Bot. 2013; 100(8): 1562–1579. 10.3732/ajb.1300039 23876453

