# Supplementary material for: Genetic structure of coast redwood (Sequoia sempervirens [D. Don] Endl.) populations in and outside of the natural distribution range based on nuclear and chloroplast microsatellite markers
Source: PLoS One. 2020 Dec 11;15(12):e0243556. doi: 10.1371/journal.pone.0243556 (PMC7732113; doi:10.1371/journal.pone.0243556)

S1 Fig. Map of the original 'Kuser's samples' (data set F). Original locations of the 'Kuser's' samples in the data set F are presented on the map together with the mean monthly temperature pattern for the time period 1979-2013 indicating colder and warmer temperatures by darker and lighter shades of grey (http://chelsa-climate.org).

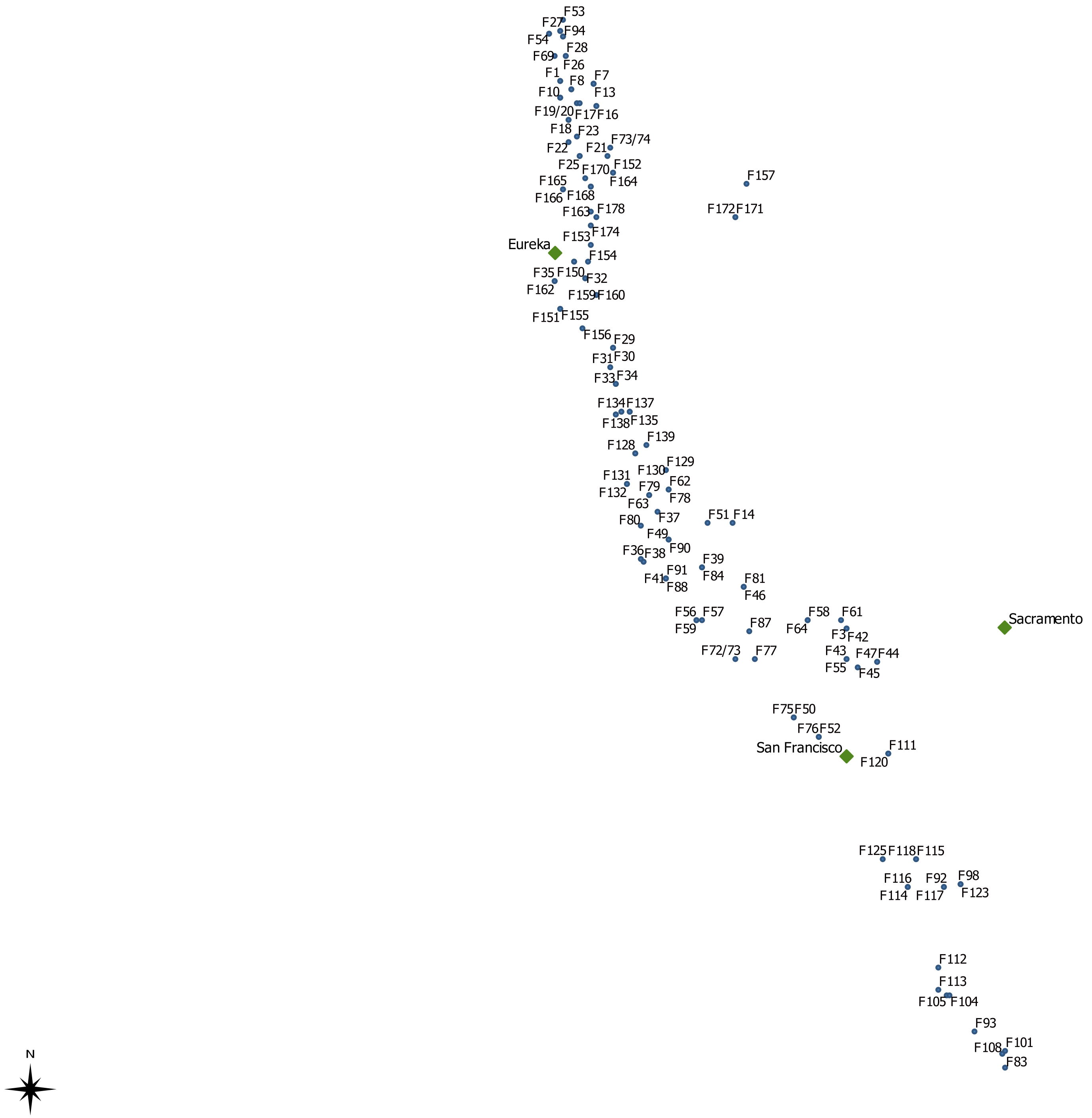

Supplement: S1 Fig — Original locations of the “Kuser’s” samples in the data set F are presented on the map together with the mean monthly temperature pattern for the time period 1979–2013 indicating colder and warmer temperatures by darker and lighter shades of grey (http://chelsa-climate.org). (PDF) [file pone.0243556.s001.pdf]
