# Supplementary material for: Genetic structure of coast redwood (Sequoia sempervirens [D. Don] Endl.) populations in and outside of the natural distribution range based on nuclear and chloroplast microsatellite markers
Source: PLoS One. 2020 Dec 11;15(12):e0243556. doi: 10.1371/journal.pone.0243556 (PMC7732113; doi:10.1371/journal.pone.0243556)

S2 Fig. Map of the German sampling locations (data set G). Darker and lighter shades of grey highlight colder and warmer mean monthly temperatures for the period 1979-2013 (http://chelsa-climate.org).

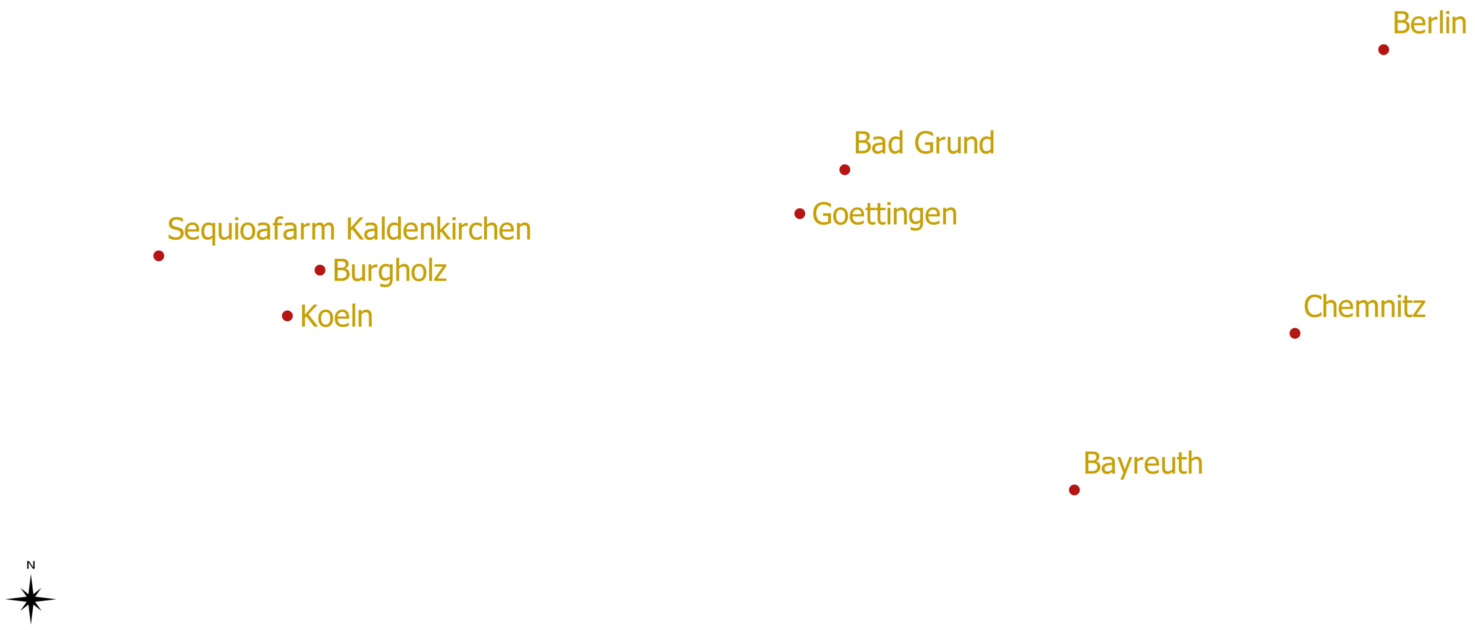

Supplement: S2 Fig — Darker and lighter shades of grey highlight colder and warmer mean monthly temperatures for the time period 1979–2013 (http://chelsa-climate.org). (PDF) [file pone.0243556.s002.pdf]
