# Supplementary material for: Genetic structure of coast redwood (Sequoia sempervirens [D. Don] Endl.) populations in and outside of the natural distribution range based on nuclear and chloroplast microsatellite markers
Source: PLoS One. 2020 Dec 11;15(12):e0243556. doi: 10.1371/journal.pone.0243556 (PMC7732113; doi:10.1371/journal.pone.0243556)

**S5 Fig. Neighbour-joining tree of the 16 Californian reference populations represented by samples collected in 2017 (data set C).** It is based on six cpSSR markers and Nei's genetic distance ([47] after [48]) with 1000 bootstraps. Numbers indicate bootstrap values in terms of percentage. County names of sampling sites are in brackets.

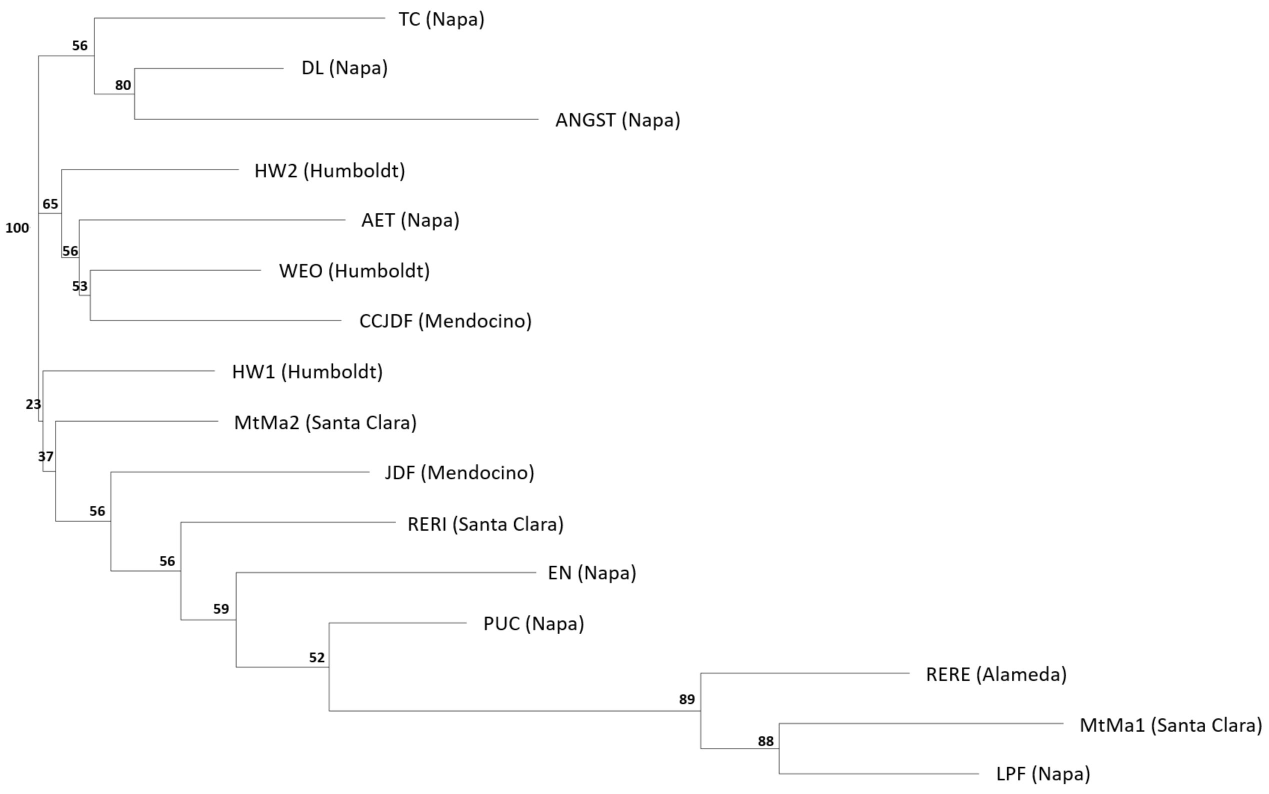

Supplement: S5 Fig — It is based on six cpSSR markers and Nei’s genetic distance ([50] after [51]) with 1000 bootstraps. Numbers indicate bootstrap values in terms of percentage. County names of sampling sites are in brackets. (PDF) [file pone.0243556.s005.pdf]
