# Supplementary material for: Genetic structure of coast redwood (Sequoia sempervirens [D. Don] Endl.) populations in and outside of the natural distribution range based on nuclear and chloroplast microsatellite markers
Source: PLoS One. 2020 Dec 11;15(12):e0243556. doi: 10.1371/journal.pone.0243556 (PMC7732113; doi:10.1371/journal.pone.0243556)

S6 Fig. Neighbour-joining tree of the 143 individuals collected in Germany (data set G). It is based on 12 nSSR markers with the final ranking combination obtained according to Pfeiffer et al. [42] and the 'bruvo genetic distance' [45] with 1000 bootstraps. Numbers indicate bootstrap values (not percentage).

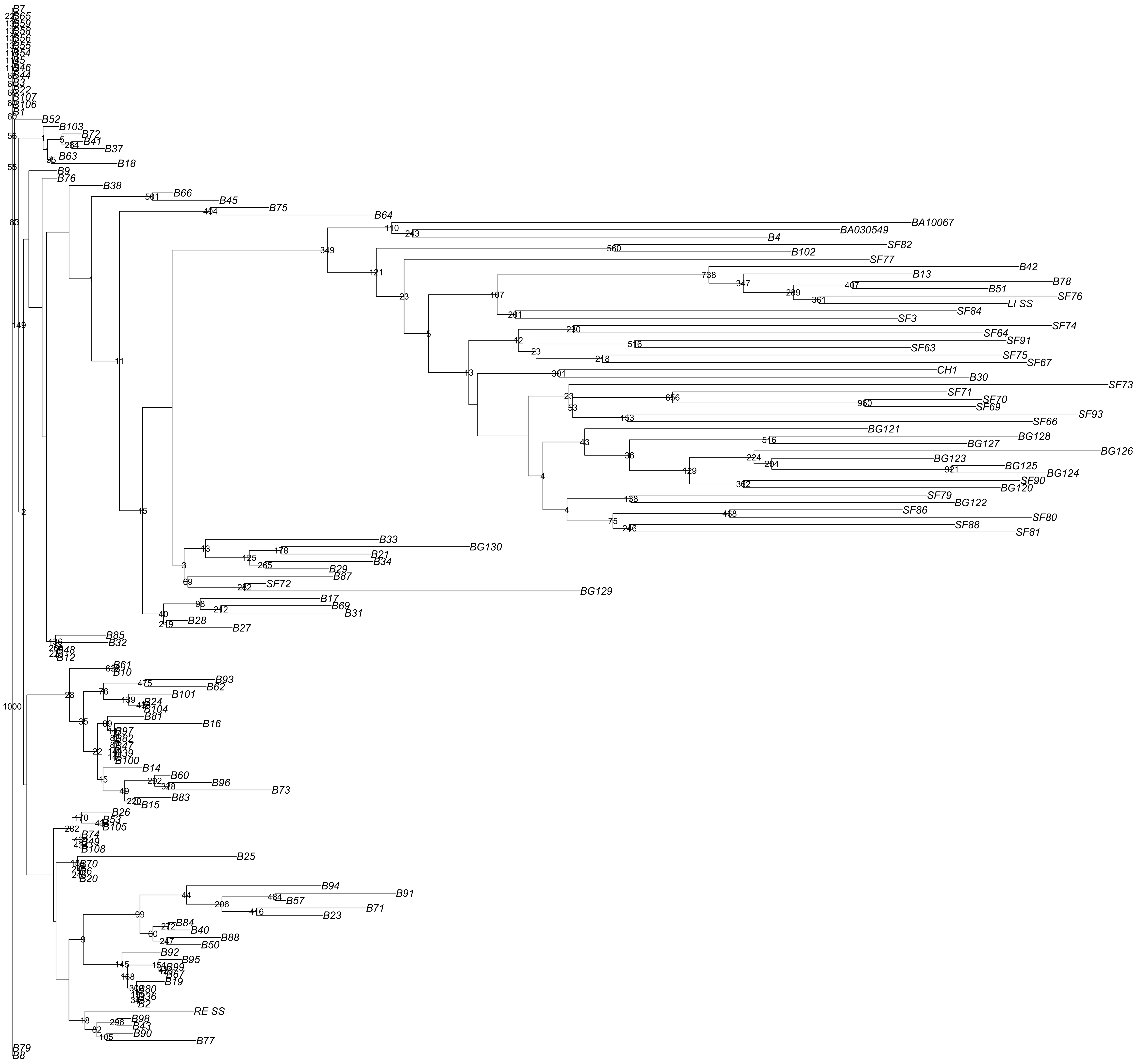

Supplement: S6 Fig — It is based on 12 nSSR markers with the final ranking combination obtained according to Pfeiffer et al. [45] and the ‘bruvo genetic distance’ [48] with 1000 bootstraps. Numbers indicate bootstrap values (not percentage). (PDF) [file pone.0243556.s006.pdf]
