# Supplementary material for: Genetic structure of coast redwood (Sequoia sempervirens [D. Don] Endl.) populations in and outside of the natural distribution range based on nuclear and chloroplast microsatellite markers
Source: PLoS One. 2020 Dec 11;15(12):e0243556. doi: 10.1371/journal.pone.0243556 (PMC7732113; doi:10.1371/journal.pone.0243556)
Supplement: S7 Fig — Plots for data sets C and F are presented for the most likely number of clusters (K) inferred without (a) and with (b) the “locprior” function engaged (K = 6 and K = 2 for C and K = 2 for F, respectively). L(K) and ΔK statistics generated by the ClumPAK software are also presented. (PDF) [file pone.0243556.s007.pdf]

S7 Fig. STRUCTURE results for the reference data sets C and F based on 12 nSSR markers. Plots for data sets C and F are presented for the most likely number of clusters (K) inferred without (a) and with (b) the 'locprior' function (K=6 and K=2 for C and K=2 for F, respectively). L(*K)* and ΔK statistics generated by the ClumPAK software are also presented.

C (a)

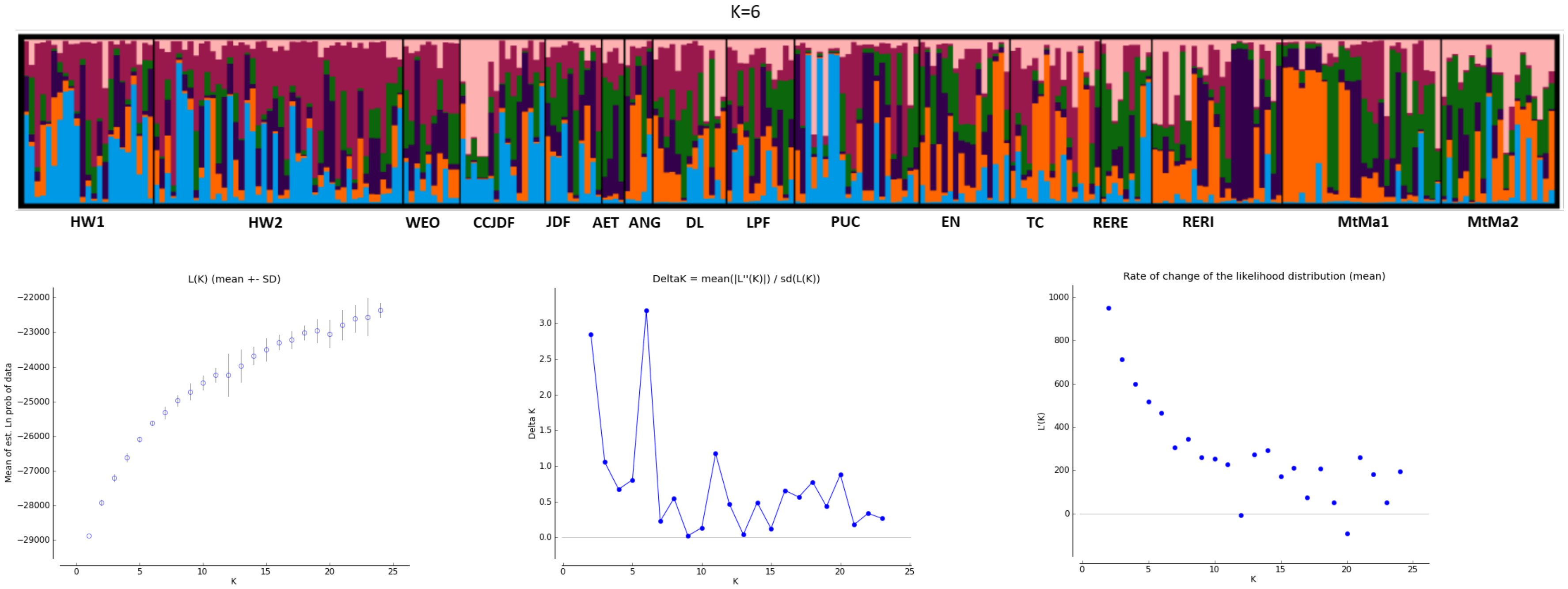

C (b)

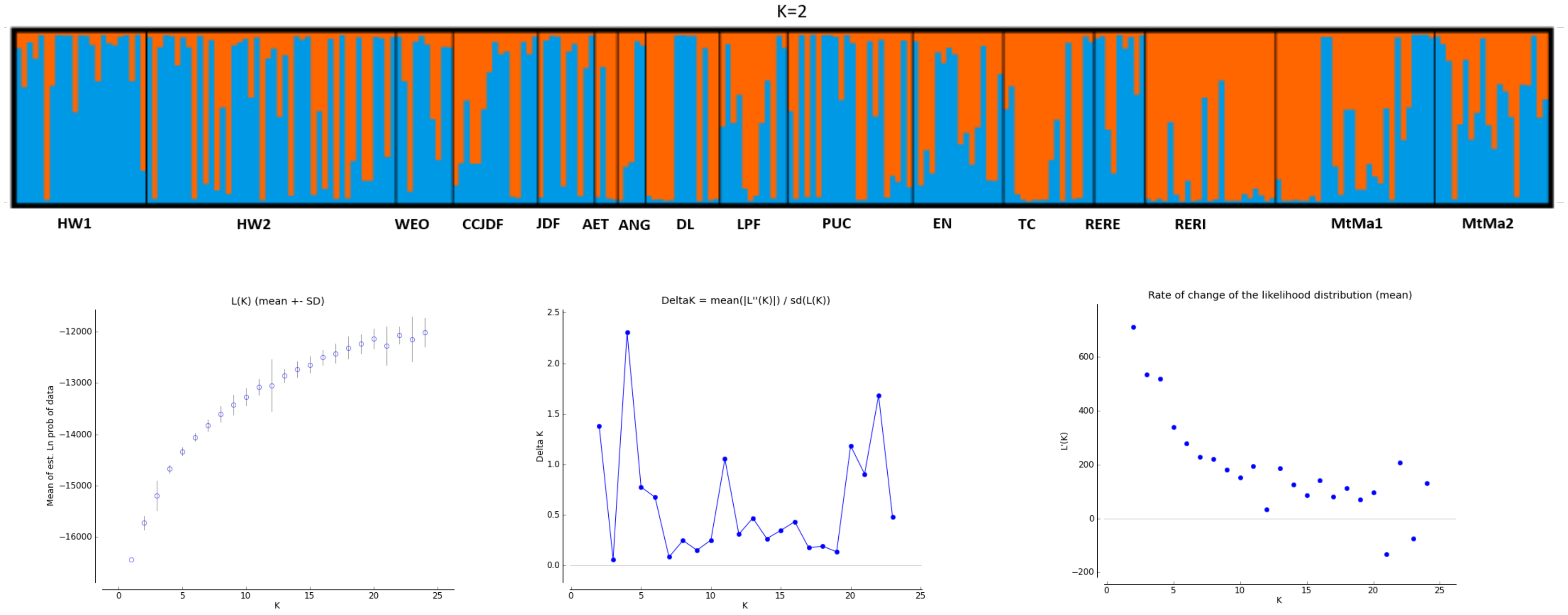

F (a)

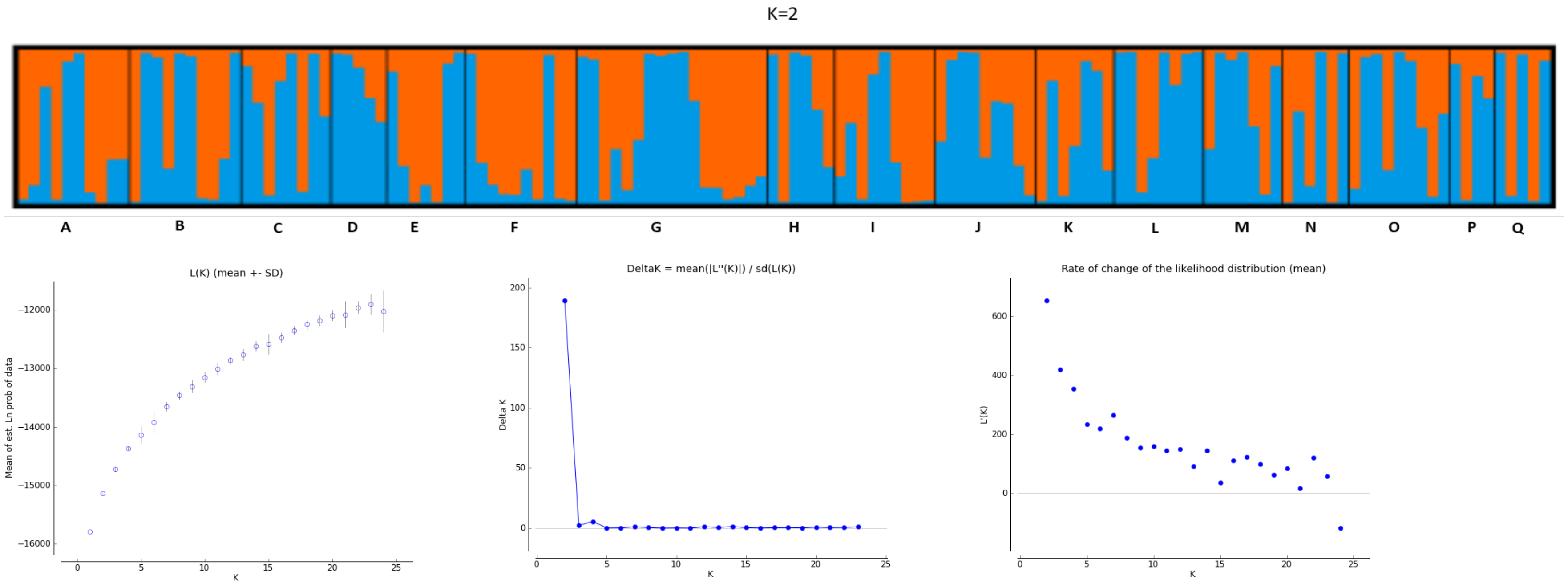
K=2
A B C D E F G H I J K L M N O P Q
L(K) (mean +- SD)
Mean of est. Ln prob of data
K
DeltaK = mean(|L''(K)|) / sd(L(K))
Delta K
K
Rate of change of the likelihood distribution (mean)
L'(K)
K

F (b)

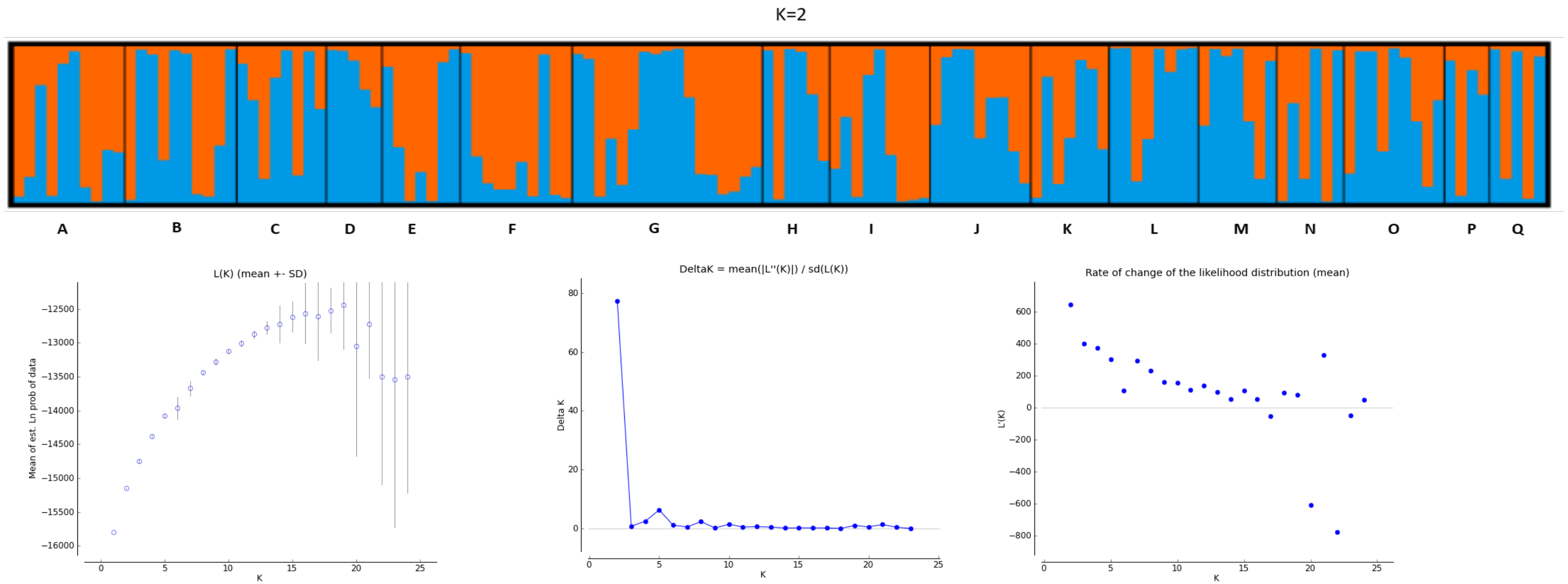
K=2
A B C D E F G H I J K L M N O P Q
L(K) (mean +- SD)
Mean of est. Ln prob of data
K
DeltaK = mean(|L''(K)|) / sd(L(K))
Delta K
K
Rate of change of the likelihood distribution (mean)
L'(K)
K
