# Supplementary material for: Genetic structure of coast redwood (Sequoia sempervirens [D. Don] Endl.) populations in and outside of the natural distribution range based on nuclear and chloroplast microsatellite markers
Source: PLoS One. 2020 Dec 11;15(12):e0243556. doi: 10.1371/journal.pone.0243556 (PMC7732113; doi:10.1371/journal.pone.0243556)

S8 Fig. STRUCTURE statistics for the reference data sets C and F based on 6 cpSSR markers. Presented are the most likely number of clusters (K) and results of STRUCTURE runs for K from 1 to 20 or 25 with 20 iterations each for the complete data set C (a), northern subset C (b), southern subset C (c), complete data set F (d), northern subset F (e), and southern subset F (f).

C (a)

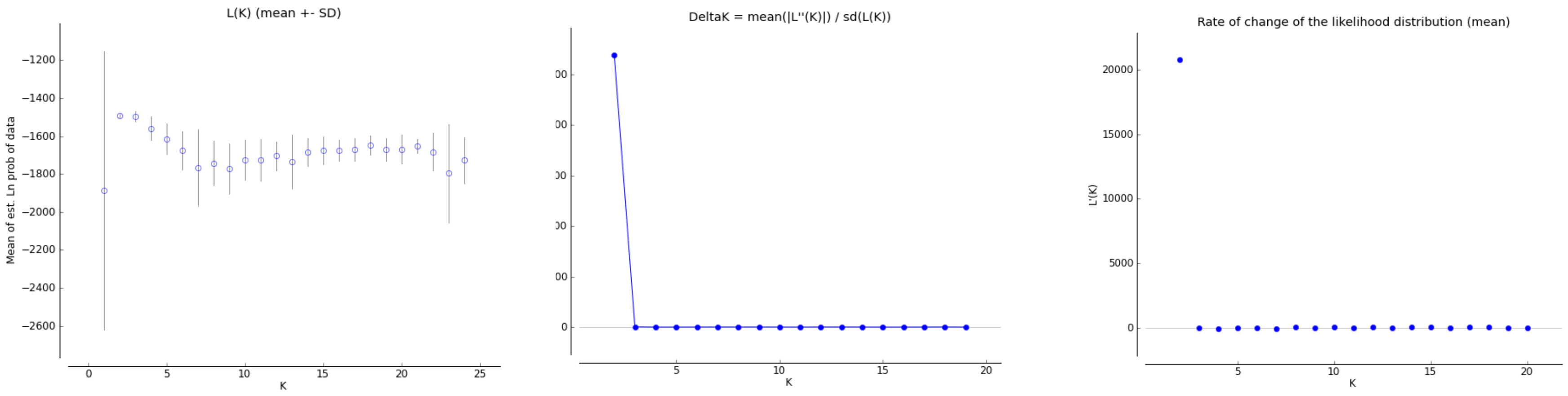

C (b)

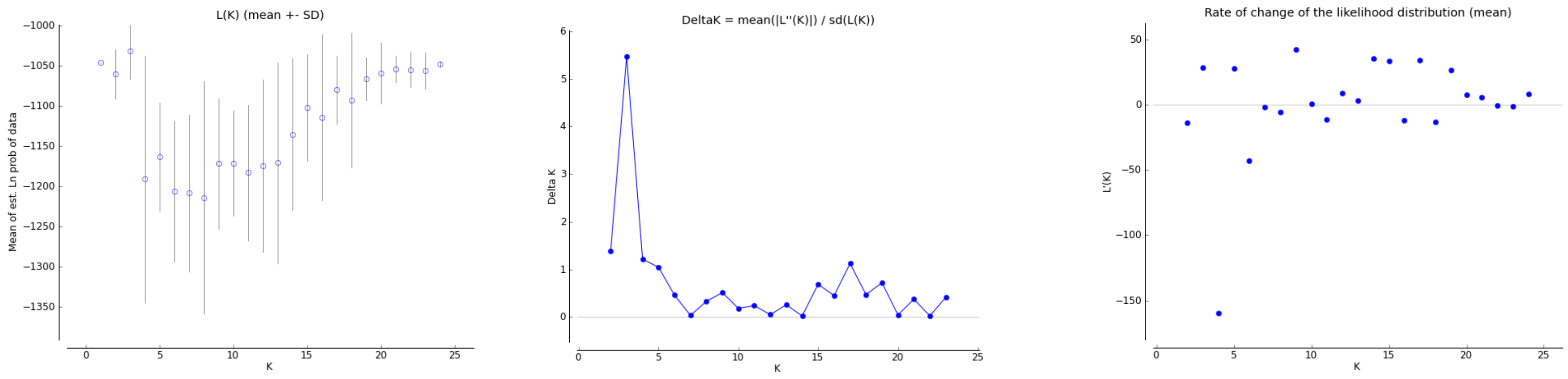

(c) southern subset of data set C, K=4

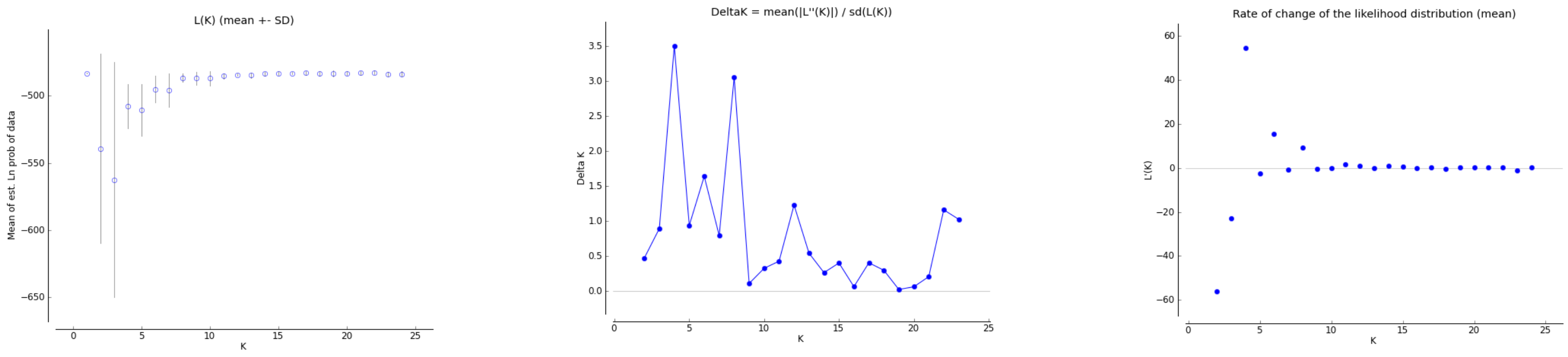

(d) complete data set F, K=2

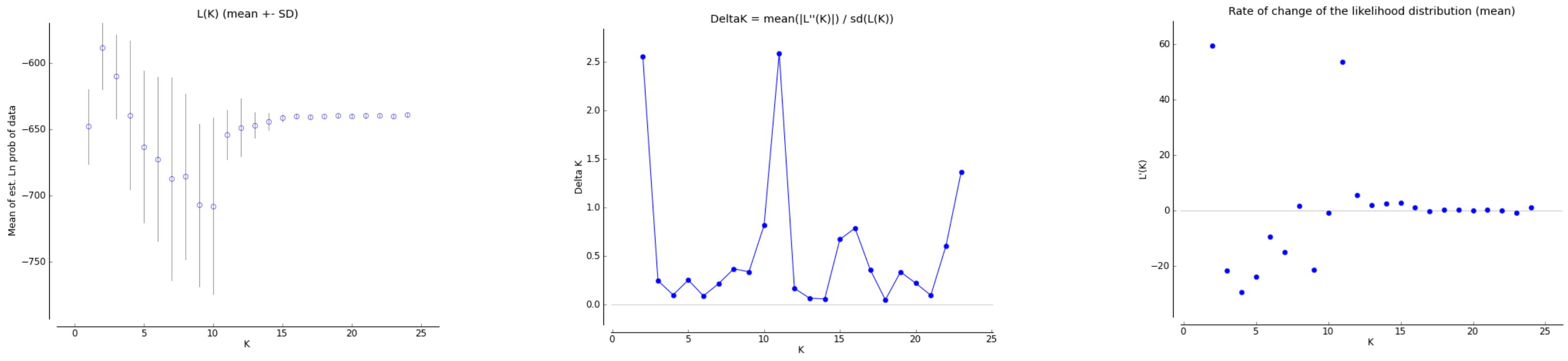

(e) northern subset of data set F, K=2

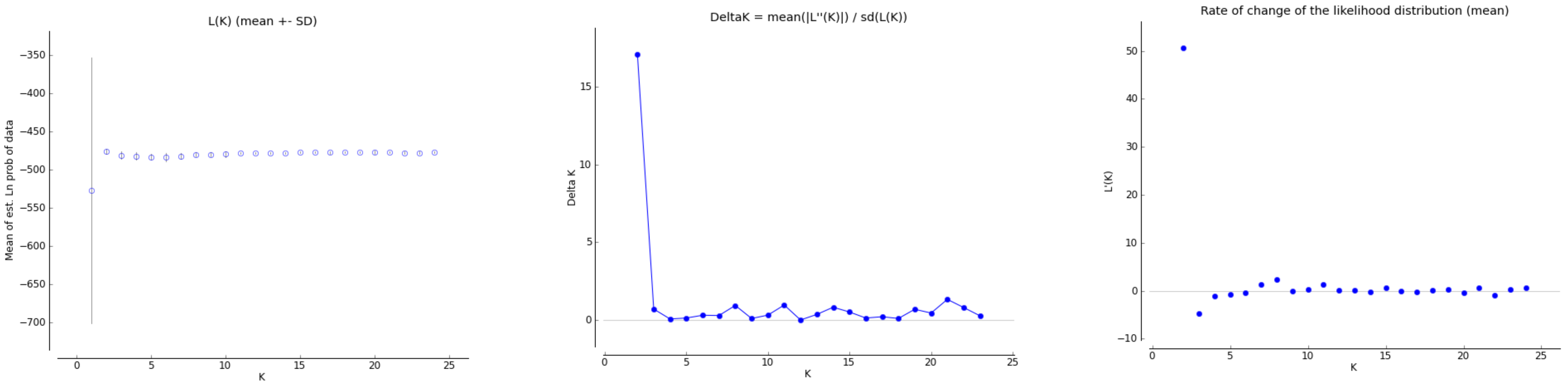

(f) southern subset of data set F, K=2

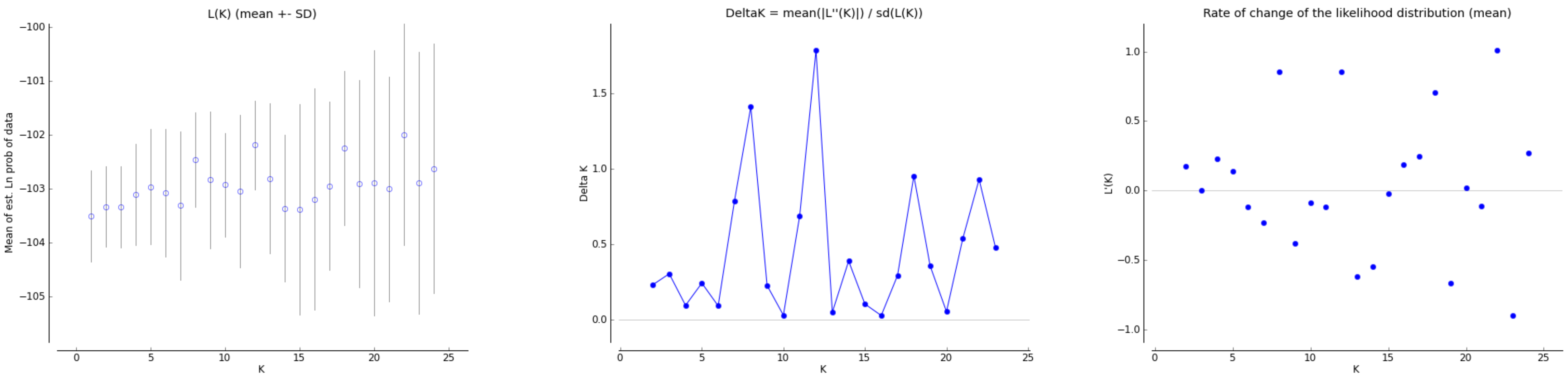

Supplement: S8 Fig — Presented are the most likely number of clusters (K) and results of STRUCTURE runs for K from 1 to 20 or 25 with 20 iterations each for the complete data set C (a), northern subset C (b), southern subset C (c), complete data set F (d), northern subset F (e), and southern subset F (f). (PDF) [file pone.0243556.s008.pdf]
