# Supplementary figures and images for: Genetic structure of coast redwood (Sequoia sempervirens [D. Don] Endl.) populations in and outside of the natural distribution range based on nuclear and chloroplast microsatellite markers
Source: PLoS One. 2020 Dec 11;15(12):e0243556. doi: 10.1371/journal.pone.0243556 (PMC7732113; doi:10.1371/journal.pone.0243556)

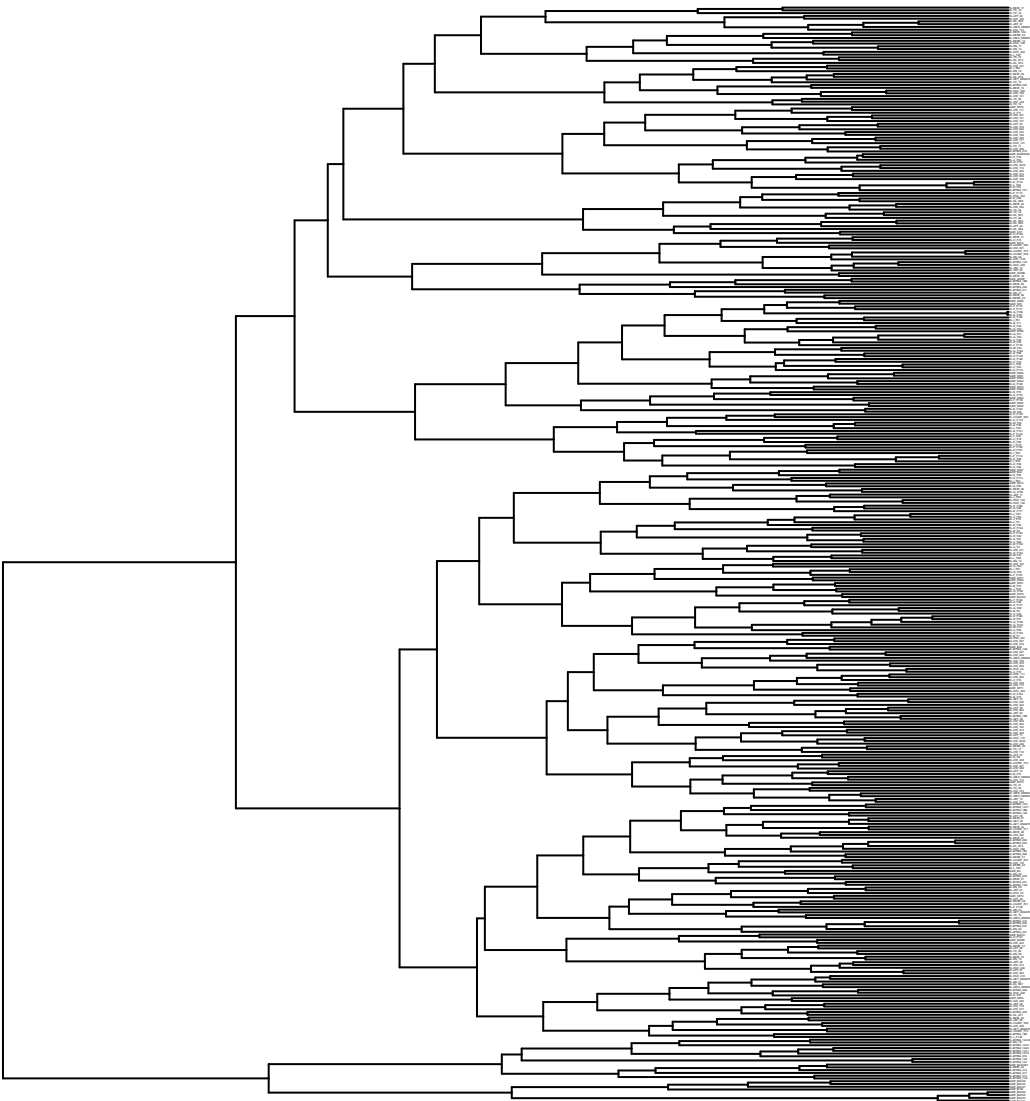

Supplement: S9 Fig — (PDF) [file pone.0243556.s009.pdf]

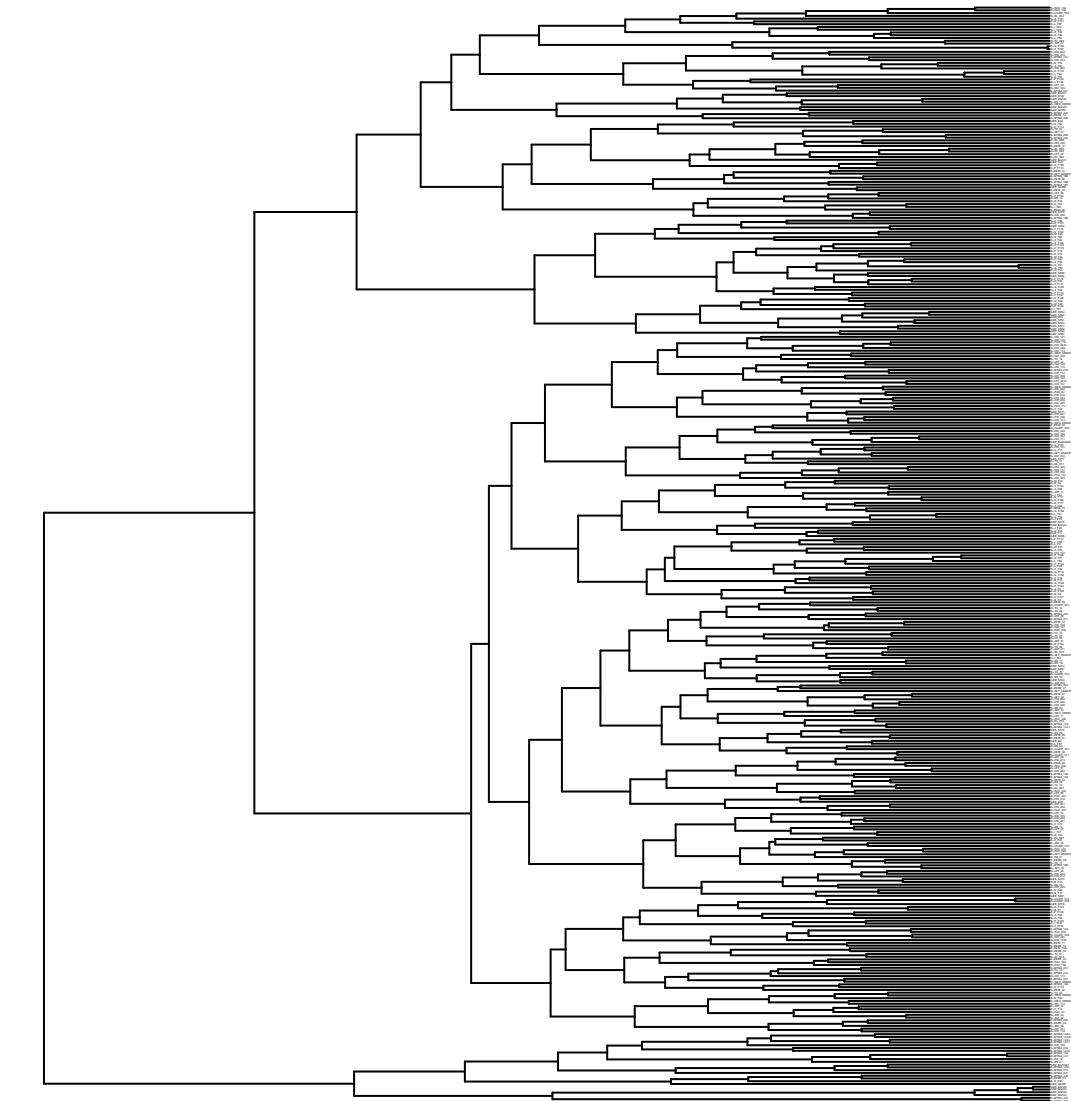

Supplement: S10 Fig — (PDF) [file pone.0243556.s010.pdf]
