# Supplementary material for: Genetic structure of coast redwood (Sequoia sempervirens [D. Don] Endl.) populations in and outside of the natural distribution range based on nuclear and chloroplast microsatellite markers
Source: PLoS One. 2020 Dec 11;15(12):e0243556. doi: 10.1371/journal.pone.0243556 (PMC7732113; doi:10.1371/journal.pone.0243556)

S12 Fig. Principal coordinate analysis (PCoA) plot based on pairwise individual tree genetic distances between all 396 trees in German (G), French (F) and Californian (C) data sets generated using *Q*-values for *K* = 30.

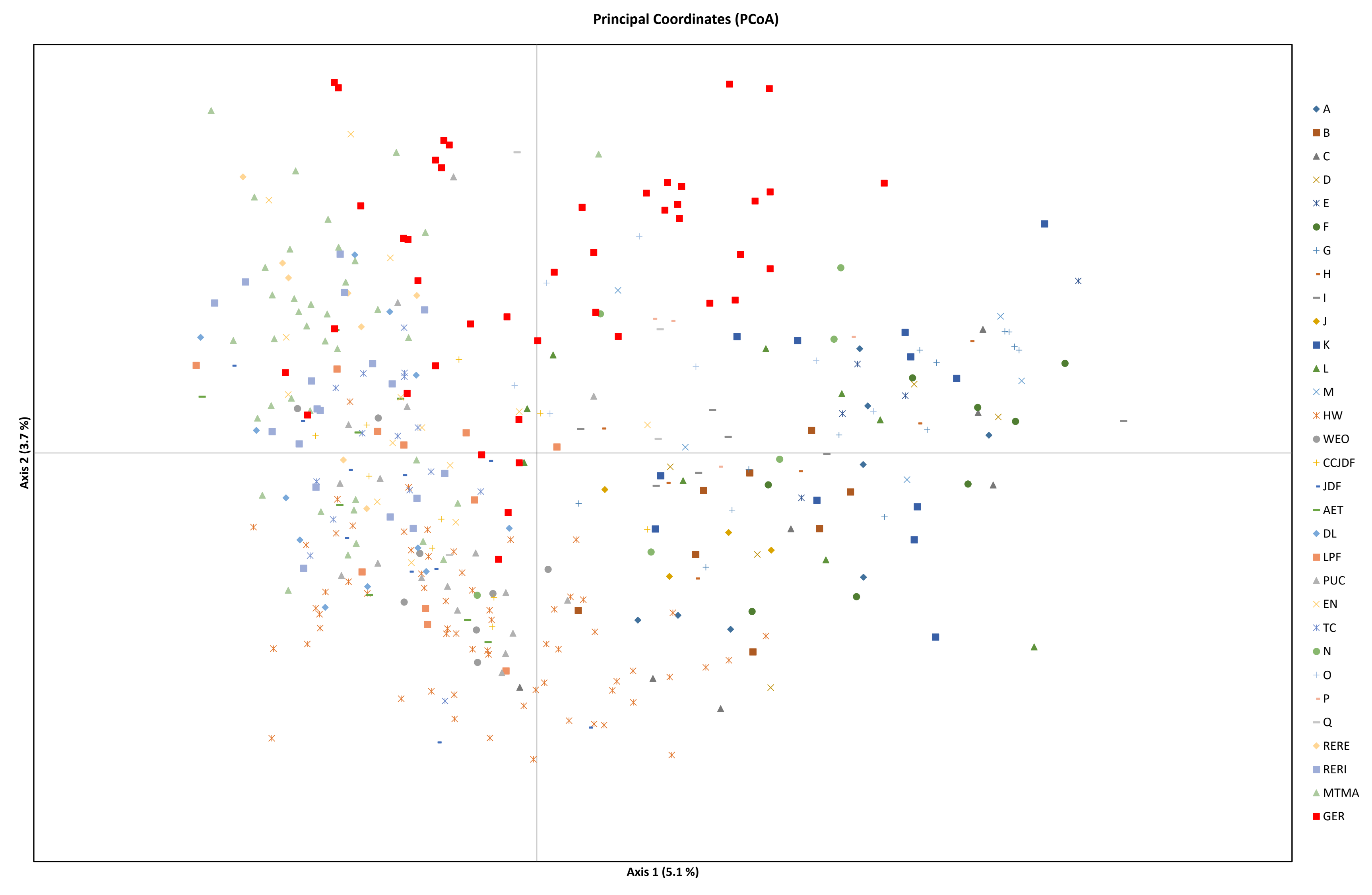

Supplement: S12 Fig — (PDF) [file pone.0243556.s012.pdf]
