# Supplementary material for: Genetic structure of coast redwood (Sequoia sempervirens [D. Don] Endl.) populations in and outside of the natural distribution range based on nuclear and chloroplast microsatellite markers
Source: PLoS One. 2020 Dec 11;15(12):e0243556. doi: 10.1371/journal.pone.0243556 (PMC7732113; doi:10.1371/journal.pone.0243556)
Supplement: S1 Table — (DOCX) [file pone.0243556.s014.docx]

**S1 Table. ID, geographic, and watershed data for the French (F) set of the “Kuser’s” samples.**

| **St. Fargeau ID** | **Kuser ID** | **Latitude** | **Longitude** | **Altitude** | **Location** | **County** | **Watershed** |
| --- | --- | --- | --- | --- | --- | --- | --- |
| F1 | D-08-2 | 41.8333333 | -124.1333333 | 24 | Wonder Stump Road | Del Norte | A |
| F3 | N-01-5 | 38.55 | -122.4166667 | 488 | Las Posadas S.F. | Sonoma | L |
| F4 | C-02-1 | 42.1333333 | -124.1333333 | 415 | Redwood Creek | Del Norte | A |
| F6 | D-03-5 | 41.8166667 | -123.9333333 | 686 | French Hill Road | Del Norte | B |
| F7 | D-03-2 | 41.8166667 | -123.9333333 | 686 | French Hill Road | Del Norte | B |
| F8 | D-02-2 | 41.7833333 | -124.0666667 | 37 | Stout Grove | Del Norte | B |
| F9 | D-02-3 | 41.7833333 | -124.0666667 | 37 | Stout Grove | Del Norte | B |
| F10 | D-04-5 | 41.7333333 | -124.1333331 | 183 | Crescent City | Del Norte | B |
| F11 | D-04-1 | 41.7 | -124.0333328 | 579 | Rock Creek | Del Norte | B |
| F12 | D-04-6 | 41.7333333 | -124.1333331 | 183 | Crescent City | Del Norte | B |
| F13 | D-03-1 | 41.8166667 | -123.9333333 | 701 | French Hill Road | Del Norte | B |
| F14 | M-10-1 | 39.1833333 | -123.0999992 | 244 | Ukiah | Mendocino | H |
| F15 | D-04-2 | 41.7 | -124.0166667 | 579 | Rock Creek | Del Norte | B |
| F16 | D-03-6 | 41.6833333 | -123.9166667 | 232 | Big Flat, S.Fk.Smith R. | Del Norte | C |
| F17 | D-03-7 | 41.6833333 | -123.9166667 | 232 | Big Flat, S.Fk.Smith R. | Del Norte | C |
| F18 | D-05-4 | 41.6 | -124.0833333 | 305 | High Prairie Creek | Del Norte | C |
| F19 | D-05-3 | 41.6 | -124.0833333 | 305 | High Prairie Creek | Del Norte | C |
| F21 | H-02-7 | 41.3833333 | -123.85 | 762 | P-1-M Road, Pecwan | Humboldt | D |
| F22 | D-06-3 | 41.4666667 | -124.0833333 | 396 | Potato Patch Creek | Del Norte | C |
| F23 | D-07-3 | 41.5 | -124.0333333 | 213 | Waukell Creek | Del Norte | C |
| F24 | H-01-3 | 41.3833333 | -124.0166667 | 61 | Prairie Creek | Humboldt | D |
| F25 | H-01-4 | 41.3833333 | -124.0166667 | 61 | Prairie Creek | Humboldt | D |
| F26 | D-01-5 | 41.9833333 | -124.1 | 457 | Winchuck R.,S.Fk | Del Norte | A |
| F27 | C-02-2 | 42.1333333 | -124.1333333 | 415 | Redwood Creek | Del Norte | A |
| F28 | D-01-8 | 41.9833333 | -124.1 | 152 | Winchuck R.,S.Fk | Del Norte | A |
| F29 | H-15-2 | 40.2333333 | -123.8166667 | 101 | Miranda | Humboldt | G |
| F30 | H-15-1 | 40.2333333 | -123.8166667 | 91 | Miranda | Humboldt | G |
| F31 | H-22-2 | 40.1166667 | -123.8333333 | 91 | Redway | Humboldt | G |
| F32 | H-20-1 | 40.65 | -123.9833333 | 356 | Lawrence Creek | Humboldt | G |
| F33 | H-18-1 | 40.0166667 | -123.8 | 142 | Richardson Grove | Humboldt | G |
| F34 | H-18-3 | 40.0166667 | -123.8 | 140 | Richardson Grove | Humboldt | G |
| F35 | H-17-2 | 40.6333333 | -124.1666667 | 146 | Fernbridge | Humboldt | G |
| F36 | M-18-2 | 38.9666667 | -123.65 | 634 | Mountain View | Mendocino | K |
| F37 | M-17-3 | 39.25 | -123.55 | 244 | Comptche | Mendocino | I |
| F38 | M-14-2 | 38.95 | -123.6333333 | 207 | Manchester | Mendocino | J |
| F39 | M-09-2 | 38.9166667 | -123.2833333 | 268 | Ornbaum Valley | Mendocino | K |
| F40 | M-19-2 | 38.85 | -123.5 | 654 | Gualala Peak | Mendocino | K |
| F41 | M-08-2 | 38.85 | -123.5 | 427 | Gualala Peak | Mendocino | K |
| F42 | N-01-4 | 38.55 | -122.4166667 | 488 | Las Posadas S.F. | Sonoma | L |
| F43 | N-03-3 | 38.3666667 | -122.4166667 | 518 | Lokoya | Napa | M |
| F44 | N-05-2 | 38.35 | -122.2333333 | 140 | Napa (E) | Napa | M |
| F45 | N-03-1 | 38.3166667 | -122.35 | 85 | Napa (W) | Napa | M |
| F46 | S-05-2 | 38.8 | -123.0333333 | 183 | Clover Dale | Mendocino | K |
| F47 | N-05-1 | 38.35 | -122.2333333 | 140 | Napa (E) | Napa | M |
| F49 | M-07-2 | 39.0833333 | -123.4833333 | 91 | Hendy S.P. | Mendocino | I |
| F50 | R-02-2 | 38.0166667 | -122.7333333 | 46 | Taylor S.P. | Marine | N |
| F51 | M-10-2 | 39.1833333 | -123.25 | 244 | Ukiah | Mendocino | I |
| F52 | R-01-1 | 37.9 | -122.5833333 | 76 | Muir Woods | Marine | N |
| F53 | C-01-01 | 42.2 | -124.1166667 | 415 | Quail Prairie | Del Norte | A |
| F54 | C-04-2 | 42.1166667 | -124.2 | 27 | Chetco River | Del Norte | A |
| F55 | N-03-4 | 38.3666667 | -122.4166667 | 518 | Lokoya | Napa | M |
| F56 | S-01-2 | 38.6 | -123.3166667 | 183 | Kruse Rhodo Res. | Sonoma | L |
| F57 | N-02-2 | 38.6 | -123.2833333 | 488 | Ink Grade | Sonoma | L |
| F58 | N-04-2 | 38.6 | -122.65 | 219 | Calistoga | Sonoma | L |
| F59 | S-01-1 | 38.6 | -123.3166667 | 183 | Kruse Rhodo Res. | Sonoma | L |
| F61 | N-02-1 | 38.6 | -122.45 | 488 | Ink Grade | Sonoma | L |
| F62 | M-12-2 | 39.3833333 | -123.4833333 | 457 | Willits | Mendocino | J |
| F63 | M-16-3 | 39.35 | -123.6 | 110 | Dunlap | Mendocino | I |
| F64 | N-04-1 | 38.6 | -122.65 | 219 | Calistoga | Sonoma | L |
| F65 | C-04-3 | 42.1166667 | -124.2 | 27 | Chetco River | Del Norte | A |
| F68 | C-03-2 | 42.1 | -124.1166667 | 366 | Wheeler Creek | Del Norte | A |
| F69 | D-01-7 | 41.9833333 | -124.1666667 | 152 | Winchuck R.,S.Fk | Del Norte | A |
| F70 | D-08-1 | 41.8333333 | -124.1333333 | 24 | Wonder Stump Road | Del Norte | B |
| F72 | S-04-3 | 38.3666667 | -123.0833333 | 122 | Bodega | Napa | M |
| F73? | H-02-6 | 41.433333 | -123.83 | 610 | Indian Creek, Pecwan | Del Norte | C |
| F75 | R-02-4 | 38.0166667 | -122.7333333 | 46 | Taylor S.P. | Marine | N |
| F76 | R-01-2 | 37.9 | -122.5833333 | 76 | Muir woods | Marine | N |
| F77 | S-04-2 | 38.3666667 | -122.9666667 | 122 | Bodega | Mendocino | M |
| F78 | M-12-1 | 39.3833333 | -123.4833333 | 457 | Willits | Mendocino | J |
| F79 | M-16-2 | 39.35 | -123.6 | 110 | Dunlap | Mendocino | I |
| F80 | M-06-1 | 39.1666667 | -123.65 | 19 | Navarro | Mendocino | I |
| F81 | S-05-1 | 38.8 | -123.0333333 | 213 | Clover Dale | Mendocino | K |
| F83 | Y-04-1 | 35.9166667 | -121.4666667 | 152 | Pacific Valley, Shruby | Monterey | Q |
| F84 | M-09-1 | 38.9166667 | -123.2833333 | 268 | Ornbaum Valley | Mendocino | K |
| F87 | S-02-2 | 38.5333333 | -123 | 34 | Armstrong Woods | Sonoma | L |
| F88 | M-19-1 | 38.85 | -123.5 | 654 | Gualala Peak | Mendocino | K |
| F89 | M-13-5 | 39.333333 | -123.25 | 335 | Redwood Valley | Mendocino | I |
| F90 | M-07-1 | 39.0833333 | -123.4833333 | 91 | Hendy S.P. | Mendocino | I |
| F91 | M-08-1 | 38.85 | -123.5 | 427 | Gualala Peak | Mendocino | K |
| F92 | Z-04-2 | 37 | -121.8333333 | 152 | Corralitos Creek | Santa Cruz | O |
| F93 | Y-01-2 | 36.1333333 | -121.65 | 116 | J.Pfeiffer Burns S.P. | Monterey | Q |
| F94 | C-03-1 | 42.1 | -124.1166667 | 366 | Wheeler Creek | Del Norte | A |
| F98 | L-01-4 | 37.0166667 | -121.7333333 | 597 | Mt Madonna | Santa Cruz | O |
| F101 | Y-03-2 | 36.0166667 | -121.4666667 | 823 | Nacimiento Road | Monterey | Q |
| F104 | Y-10-1 | 36.35 | 178 | 585 | Bottcher’s Gap | Monterey | P |
| F105 | Y-10-3 | 36.35 | -121.8166667 | 585 | Bottcher’s Gap | Monterey | P |
| F106 | Y-03-4 | 36 | -121.4833333 | 518 | Nacimiento Road | Monterey | Q |
| F108 | Y-03-6 | 36 | -121.4833333 | 518 | Nacimiento Road | Monterey | Q |
| F111 | A-01-6 | 37.8 | -122.1666667 | 259 | Redwood Regional Park | Marine | N |
| F112 | Y-09-1 | 36.5166667 | -121.8666667 | 49 | Robinson Canyon | Monterey | P |
| F113 | Y-08-2 | 36.3833333 | -121.8666664 | 274 | Palo Colorado Rd. | Monterey | P |
| F114 | Z-02-6 | 37 | -122.05 | 213 | U.C.Santa Cruz | Santa Cruz | O |
| F115 | Z-03-1 | 37.1666667 | -122 | 463 | Zayante Creek | Santa Cruz | O |
| F116 | Z-02-4 | 37 | -122.05 | 213 | U.C.Santa Cruz | Santa Cruz | O |
| F117 | Z-04-1 | 37 | -121.8333333 | 152 | Corralitos Creek | Santa Cruz | O |
| F118 | Z-03-2 | 37.1666667 | -122 | 463 | Zayante creek | Santa Cruz | O |
| F120 | A-01-5 | 37.8 | -122.1666667 | 259 | Redwood Regional Park | Marine | N |
| F123 | L-01-2 | 37.0166667 | -121.7333333 | 526 | Mt Madonna | Santa Cruz | O |
| F125 | Z-01-2 | 37.1666667 | -122.2 | 381 | Big Basin S.P. | Santa Cruz | O |
| F127 | M-04-1 | 39.6 | -123.6833333 | 61 | Ten Mile R.,N.Fk. | Mendocino | I |
| F128 | M-04-3 | 39.6 | -123.6833333 | 61 | Ten Mile R.,N.Fk. | Mendocino | K |
| F129 | M-11-1 | 39.5 | -123.5 | 805 | Sherwood Peak | Mendocino | K |
| F130 | M-11-2 | 39.5 | -123.5 | 774 | Sherwood Peak | Mendocino | K |
| F131 | M-05-1 | 39.4166667 | -123.7333333 | 195 | Noyo River S.Fk. | Mendocino | K |
| F132 | M-05-2 | 39.4166667 | -123.7333333 | 195 | Noyo River S.Fk. | Mendocino | K |
| F133 | M-02-1 | 39.85 | -123.7666667 | 549 | Hale´s Grove | Humboldt | G |
| F134 | M-02-2 | 39.85 | -123.7666667 | 549 | Hale´s Grove | mendocino | H |
| F135 | M-01-4 | 39.85 | -123.7166667 | 335 | Leggett | mendocino | H |
| F136 | M-03-2 | 39.8333333 | -123.8 | 61 | Usal Creek | mendocino | H |
| F137 | M-01-5 | 39.85 | -123.7166667 | 335 | Leggett | mendocino | H |
| F138 | M-03-3 | 39.8333333 | -123.8 | 61 | Usal Creek | mendocino | H |
| F139 | M-15-1 | 39.65 | -123.6166667 | 482 | Branscomb | Mendocino | J |
| F140 | M-15-2 | 39.65 | -123.6166667 | 482 | Branscomb | Mendocino | I |
| F149 | H-04-2 | 41.1833333 | -124.1166667 | 30 | Big Lagoon | Humboldt | E |
| F150 | H-20-2 | 40.65 | -123.9833333 | 356 | Lawrence Creek | Humboldt | F |
| F151 | H-14-3 | 40.4666667 | -124.1333333 | 503 | Rio Dell | Humboldt | G |
| F152 | H-02-4 | 41.2833333 | -123.8166667 | 76 | Cappel Creek | Humboldt | D |
| F153 | H-09-5 | 40.85 | -123.95 | 122 | Mad River S.Fk | Humboldt | F |
| F154 | H-11-5 | 40.75 | -123.9666667 | 684 | Kneeland | Humboldt | F |
| F155 | H-14-4 | 40.4666667 | -124.1333333 | 457 | Rio Dell | Humboldt | G |
| F156 | H-16-2 | 40.35 | -124 | 91 | Bull Creek | Humboldt | G |
| F157 | H-08-2 | 41.2166667 | -123.0166667 | 30 | Tall Trees, Redwood Creek | Humboldt | E |
| F159 | H-12-1 | 40.55 | -123.9166667 | 762 | Yager Creek | Humboldt | G |
| F160 | H-12-2 | 40.55 | -123.9166667 | 762 | Yager creek | Humboldt | G |
| F162 | H-17-1 | 40.6333333 | -124.1666667 | 146 | Fernbridge | Humboldt | G |
| F163 | H-05-4 | 41.05 | -123.95 | 419 | Little River | Humboldt | F |
| F164 | H-02-5 | 41.2833333 | -123.8166667 | 91 | Cappel Creek | Humboldt | D |
| F165 | H-04-1 | 41.1833333 | -124.1166667 | 30 | Big Lagoon | Humboldt | E |
| F166 | H-04-2 | 41.1833333 | -124.1166667 | 30 | Big Lagoon | Humboldt | E |
| F167 | H-11-4 | 40.75 | -124.05 | 30 | Freshwater | Humboldt | F |
| F168 | H-03-3 | 41.2 | -123.95 | 716 | Roach Creek | Humboldt | E |
| F170 | H-08-4 | 41.25 | -123.9833333 | 579 | Tall Trees, Redwood Creek | Humboldt | E |
| F171 | H-07-4 | 41.0166667 | -123.0833333 | 146 | Crannell | Humboldt | F |
| F172 | H-07-3 | 41.0166667 | -123.0833333 | 146 | Crannell | Humboldt | F |
| F173 | H-11-3 | 40.75 | -124.05 | 30 | Freshwater | Humboldt | F |
| F174 | H-10-4 | 40.9666667 | -123.95 | 244 | The Basin | Humboldt | F |
| F175 | H-13-1 | 40.483333 | -123.916667 | 137 | Grizzly Creek | Humboldt | G |
| F176 | H-13-3 | 40.483333 | -123.916667 | 137 | Grizzly Creek | Humboldt | G |
| F177 | H-03-4 | 41.2 | -123.95 | 625 | Roach Creek | Humboldt | E |
| F178 | H-06-3 | 41.0166667 | -123.9166667 | 671 | Wiregrass Ridge | Humboldt | F |
| F179 | H-17-2 | 40.6333333 | -124.1666667 |  | Fernbridge | Humboldt | C |
| F180 | H-08-4 | 41.25 | -123.9833333 | 579 | Tall Trees, Redwood Creek | Humboldt | E |
| F181 | H-20-1 | 40.65 | -123.9833333 | 356 | Lawrence Creek | Humboldt | G |
| F182 | H-04-1 | 41.1833333 | -124.1166667 | 30 | Big Lagoon | Humboldt | E |

Watershed information is presented in S1 Table.
