# Supplementary material for: Genetic structure of coast redwood (Sequoia sempervirens [D. Don] Endl.) populations in and outside of the natural distribution range based on nuclear and chloroplast microsatellite markers
Source: PLoS One. 2020 Dec 11;15(12):e0243556. doi: 10.1371/journal.pone.0243556 (PMC7732113; doi:10.1371/journal.pone.0243556)
Supplement: S2 Table — (DOCX) [file pone.0243556.s015.docx]

**S2 Table. Original latitude and number of samples representing 17 watersheds in the French (F) data set (St. Fargeau) and the Russell Reserve according to Douhovnikoff and Dodd [34].**

| **Watershed** | **Number of samples** | | **Original latitude** | |
| --- | --- | --- | --- | --- |
|  | **Russell reserve** | **St. Fargeau** | **Russell reserve** | **St. Fargeau** |
| **A** | 9 | 8 | 42.08 | 42.07 |
| **B** | 11 | 8 | 41.76 | 41.77 |
| **C** | 9 | 7 | 41.4 | 41.57 |
| **D** | 4 | 5 | 41.28 | 41.34 |
| **E** | 5 | 5 | 41.22 | 41.21 |
| **F** | 9 | 8 | 40.98 | 40.93 |
| **G** | 21 | 13 | 40.4 | 40.43 |
| **H** | 4 | 6 | 39.84 | 39.84 |
| **I** | 7 | 7 | 39.37 | 39.19 |
| **J** | 8 | 8 | 39.43 | 39.50 |
| **K** | 13 | 8 | 38.97 | 38.87 |
| **L** | 6 | 9 | 38.58 | 38.58 |
| **M** | 7 | 5 | 38.49 | 38.35 |
| **N** | 4 | 6 | 37.96 | 37.91 |
| **O** | 8 | 7 | 37.05 | 37.07 |
| **P** | 4 | 4 | 36.35 | 36.40 |
| **Q** | 6 | 4 | 36.00 | 36.02 |
