# Supplementary material for: Genetic structure of coast redwood (Sequoia sempervirens [D. Don] Endl.) populations in and outside of the natural distribution range based on nuclear and chloroplast microsatellite markers
Source: PLoS One. 2020 Dec 11;15(12):e0243556. doi: 10.1371/journal.pone.0243556 (PMC7732113; doi:10.1371/journal.pone.0243556)
Supplement: S3 Table — (DOCX) [file pone.0243556.s016.docx]

**S3 Table. Geographic and climatic data for sampled locations in California (data set C).**

| **Location** | **Abbreviation** | **Number of samples** | **Latitude** | **Longitude** | **Altitude, m a.s.l.** | **Precipitation, mm** | **Temperature*, °C** | | |
| --- | --- | --- | --- | --- | --- | --- | --- | --- | --- |
|  |  |  |  |  |  |  | **min** | **mean** | **max** |
| Aetna spring road | AET | 6 | 38.64644397 | -122.508037 | 470 | 1032 | 7.55 | 14.78 | 22.01 |
| Angwin road | ANG | 6 | 38.60211804 | -122.451437 | 522 | 309 | 4.72 | 8.36 | 11.44 |
| Dunn Wildlake Ranch | DL | 15 | 38.62176 | -122.50173 | 506 | 1053 | 7.12 | 13.91 | 20.70 |
| Enchanted Hills | EN | 17 | 38.38398596 | -122.422102 | 281 | 983 | 7.43 | 14.45 | 21.47 |
| Headwater 1 | HW1 | 29 | 40.61923703 | -124.081475 | 443 | 1453 | 7.24 | 12.80 | 18.35 |
| Headwater 2 | HW2 | 47 | 40.62747401 | -124.100346 | 376 | 1453 | 7.24 | 12.80 | 18.35 |
| Jackson Demonstration Forest | JDF | 13 | 39.342298 | -123.489496 | 449 | 1151 | 5.66 | 13.09 | 20.53 |
| Las Posadas Forest | LPF | 12 | 38.56557797 | -122.415119 | 493 | 967 | 7.17 | 14.29 | 21.42 |
| Mt Madonna Park | MTMA1 | 35 | 37.01058799 | -121.709825 | 562 | 887 | 8.16 | 14.50 | 20.85 |
| Mt Madonna Park | MTMA2 | 22 | 37.01294398 | -121.714812 | 526 | 887 | 8.16 | 14.50 | 20.85 |
| Pacific Union College | PUC | 26 | 38.57878902 | -122.42007 | 553 | 967 | 7.17 | 14.29 | 21.42 |
| Recreational area JDF | CCJDF | 16 | 39.35394298 | -123.560602 | 176 | 1125 | 5.98 | 13.25 | 20.52 |
| Redwood Regional Park | RERE | 11 | 37.817892 | -122.176575 | 473 | 690 | 8.61 | 13.95 | 19.27 |
| Redwood Ridge Estate | RERI | 26 | 37.16097501 | -122.005626 | 675 | 1062 | 8.38 | 14.36 | 20.34 |
| The Cove | TC | 17 | 38.37867603 | -122.437441 | 550 | 983 | 7.43 | 14.45 | 21.47 |
| Weott | WE | 11 | 40.32285897 | -123.92611 | 20 | 1306 | 6.66 | 12.73 | 18.80 |

* based on the years 1895-2015 retrieved from http://www.prism.oregonstate.edu/explorer in October 2017.
