# Supplementary material for: Genetic structure of coast redwood (Sequoia sempervirens [D. Don] Endl.) populations in and outside of the natural distribution range based on nuclear and chloroplast microsatellite markers
Source: PLoS One. 2020 Dec 11;15(12):e0243556. doi: 10.1371/journal.pone.0243556 (PMC7732113; doi:10.1371/journal.pone.0243556)
Supplement: S5 Table — (DOCX) [file pone.0243556.s018.docx]

**S5 Table. Data for 18 microsatellite (SSR) markers used in the study.**

| **SSR marker [dye]** | **Type** | **Reference** | **Multiplex** | **Motif** | **Ranking*** | **Allele size range, bp** | **Number of alleles in the sets** | | | **Forward (F) and reverse (R) PCR primer nucleotide sequences** |
| --- | --- | --- | --- | --- | --- | --- | --- | --- | --- | --- |
|  |  |  |  |  |  |  | **Californian (C)** | **French (F)** | **German (G)** |  |
| *RW39* [Fam] | nSSR | Douhovnikoff | I | CTAT | 9 | 231-451 | 56 | 52 | 40 | F:CCATAAGGTTGAAATGAAGAAAAA |
|  |  | & Dodd 2011 |  |  |  |  |  |  |  | R:GTTGATTGATCGTTGGTTGG |
| *RW48* [Hex] | nSSR | Douhovnikoff | I | CTAT | 9 | 119-205 | 31 | 22 | 13 | F:GGCCTACATAAGTGAACTAGAA |
|  |  | & Dodd 2011 |  |  |  |  |  |  |  | R:TGAGGAGAACACAACCCTCA |
| *SS36782* [Fam] | EST-SSR | Breidenbach | II | GA | 8 | 173-193 | 6 | 5 | 4 | F:TCAGGGCAAAGCTAAAATCG |
|  |  | et al. 2019 |  |  |  |  |  |  |  | R:CCAGGAAAGGAAAGGGAGAG |
| *SS74800* [Hex] | EST-SSR | Breidenbach | II | TGG | 7 | 205-238 | 12 | 10 | 9 | F:GCATGACTCTGGTGGTGTTG |
|  |  | et al. 2019 |  |  |  |  |  |  |  | R:GCAGCAGCCACTGTGAATAA |
| *SS91170* [Fam] | EST-SSR | Breidenbach | III | CA | 9 | 146-202 | 12 | 12 | 9 | F:TCTGAAAAATGCCAAATCCA |
|  |  | et al. 2019 |  |  |  |  |  |  |  | R:CGTGTGTCCTGTAAGTGCAAA |
| *Seq20* [Hex] | nSSR | Bruno & | III | CA | 7 | 187-201 | 7 | 9 | 8 | F:GATGCGGTTTGGGAA |
|  |  | Brinegar 2004 |  |  |  |  |  |  |  | R:GCAAGCATTCTGACAATGAAC |
| *SS73361* [Hex] | EST-SSR | Breidenbach | IV | TC | 10 | 190-216 | 12 | 14 | 8 | F:AGGGTAGATGGGCGGTAGTT |
|  |  | et al. 2019 |  |  |  |  |  |  |  | R:CGTCCGACAAGTTCAGTACG |
| *RW56* [Fam] | nSSR | Narayan 2015 | IV | compound | 9 | 189-267 | 21 | 17 | 12 | F:CTTGACATCATCCATAGCT |
|  |  |  |  |  |  |  |  |  |  | R:AAATTGCAAGGGGTGCAA |
| *SS73307* [Hex] | EST-SSR | Breidenbach | V | CA | 9 | 208-235 | 17 | 13 | 13 | F:GAACTGTGAAAGCCCTTGGT |
|  |  | et al. 2019 |  |  |  |  |  |  |  | R:GGGCGTGTTCTGTTTGAACT |
| *SS73978* [Hex] | EST-SSR | Breidenbach | VI | TC | 7 | 214-220 | 5 | 5 | 8 | F:CCTGCAAACAATTCCAGCTT |
|  |  | et al. 2019 |  |  |  |  |  |  |  | R:AGTGGGAATTATGGGGTTGG |
| *Seq21* [Fam] | cpSSR | Bruno & | VI | CTTA |  | 93-189 | 14 | 15 | 9 | F:GCTACTCGAGCTCCATTGG |
|  |  | Brinegar 2004 |  |  |  |  |  |  |  | R:TTCGGTGTTCAGCTTTGAGC |
| *SS114481* [Fam] | EST-SSR | Breidenbach | VII | TA | 7 | 184-205 | 10 | 13 | 10 | F:GGGTCAAGCGTGGTTATTGT |
|  |  | et al. 2019 |  |  |  |  |  |  |  | R:TCTGGCATGATCCAAGTGTT |
| *Seq18* [Hex] | nSSR | Bruno & | VII | CTT | 9 | 120-187 | 20 | 20 | 12 | F:GCAAAAAGGGAATTGTAATTGGGTTCA |
|  |  | Brinegar 2004 |  |  |  |  |  |  |  | R:CCCTAGGTCTAGGCTACGCGACTTG |
| *ss40585* [Hex] | cpSSR | Breidenbach | VIII | AT |  | 269-279 | 6 | 4 | 2 | F: [M13]-TCTTTTTCTTCAAGCACTTGTTTTT |
|  |  | et al. 2019 |  |  |  |  |  |  |  | R: [PIG-TAIL]-TCAATCTACACGGGGATGTTT |
| *ss85281* [Hex] | cpSSR | Breidenbach | VIII | AT |  | 214-222 | 6 | 4 | 2 | F: [M13]-TGACCATAGGTTCCTTCCTTTTT |
|  |  | et al. 2019 |  |  |  |  |  |  |  | R: [PIG-TAIL]-TTCCGTTCCTTTCCATTTTG |
| *ss109990* [Fam] | cpSSR | Breidenbach | VIII | TA |  | 224-232 | 3 | 4 | 3 | F: [M13]-AAAAATCGACCGGATCACAA |
|  |  | et al. 2019 |  |  |  |  |  |  |  | R: [PIG-TAIL]-TTCAAATAATAGAATGGAAAAACCAA |
| *ss49836* [Fam] | cpSSR | Breidenbach | IX | AT |  | 232-250 | 10 | 9 | 5 | F: [M13]-TGAAAGCTCTCGTGCGTATT |
|  |  | et al. 2019 |  |  |  |  |  |  |  | R: [PIG-TAIL]-AGTTGAGTTCCCGGTTCTCC |
| *ss60974* [Hex] | cpSSR | Breidenbach | IX | AT |  | 221-237 | 9 | 7 | 5 | F: [M13]-GCTCCGGCGTATAGAGAGG |
|  |  | et al. 2019 |  |  |  |  |  |  |  | R: [PIG-TAIL]-GAGATTCCAATGGCTTTTGC |

*Ranking category for the nSSR genotype scoring according to Pfeiffer et al. [45].

**References**

Breidenbach N, Gailing O, Krutovsky KV. Development of novel polymorphic nuclear and chloroplast microsatellite markers in coast redwood (*Sequoia sempervirens*). Plant Genetic Resources: Characterization and Utilization. 2019; 17(3): 293-297. doi:10.1017/S147926211800045X

Bruno C, Brinegar C. Microsatellite markers in coast redwood (*Sequoia sempervirens*). Molecular Ecology Notes. 2004; 4: 482–484.

Douhovnikoff V, Dodd R. Lineage divergence in coast redwood (*Sequoia sempervirens*), detected by a new set of nuclear microsatellite loci. American Midland Naturalist. 2011; 165(1): 22–37.

Narayan L, Dodd RS, O’Hara KL. A genotyping protocol for multiple tissue types from the polyploid tree species *Sequoia sempervirens* (Cupressaceae). Applied Plant Science. 2015; 3: 1–7.
