# Supplementary material for: Genetic structure of coast redwood (Sequoia sempervirens [D. Don] Endl.) populations in and outside of the natural distribution range based on nuclear and chloroplast microsatellite markers
Source: PLoS One. 2020 Dec 11;15(12):e0243556. doi: 10.1371/journal.pone.0243556 (PMC7732113; doi:10.1371/journal.pone.0243556)
Supplement: S8 Table — (DOCX) [file pone.0243556.s021.docx]

**S8 Table. The German samples in the data set G assigned to the populations and watersheds in the reference data sets representing samples from California (C) and the “Kuser Provenance test” in St. Fargeau, central France (F), respectively, and assignment quality scores (%) for two ranks.**

| **Sample** | **Reference C** | | | | **Reference F** | | | |
| --- | --- | --- | --- | --- | --- | --- | --- | --- |
|  | **Rank 1** | | **Rank 2** | | **Rank 1** | | **Rank 2** | |
|  | **Assigned populations and watersheds (county)** | **Score, %** | **Assigned populations and watersheds (county)** | **Score, %** | **Assigned watersheds (county)** | **Score, %** | **Assigned watersheds (county)** | **Score, %** |
| GOE_P | RERI, O (Santa Clara) | 97 | RERE, N (Santa Clara) | 2 | Q (Monterey) | 55 | N (Marine) | 14 |
| B42 | JDF, J (Mendocino) | 86 | AET, M (Napa) | 6 | G (Humboldt) | 33 | C (Del Norte) | 26 |
| SF85 | JDF, J (Mendocino) | 86 | AET, M (Napa) | 6 | G (Humboldt) | 33 | C (Humboldt) | 26 |
| BA10067 | MtMa, O (Santa Clara) | 85 | LPF, M (Napa) | 6 | O (Santa Cruz) | 60 | N (Marine) | 15 |
| GOE_B | MtMa, O (Santa Clara) | 65 | RERI, O (Santa Clara) | 15 | P (Monterey) | 69 | K (Mendocino) | 18 |
| SF65 | WEO, G (Humboldt) | 59 | HW, G (Humboldt) | 14 | E (Humboldt) | 30 | I (Mendocino) | 27 |
| SF80 | AET, M (Napa) | 58 | JDF, J (Mendocino) | 16 | J (Mendocino) | 39 | F (Humboldt) | 32 |
| SF75 | JDF, J (Mendocino) | 58 | TC, M (Napa) | 30 | J (Mendocino) | 25 | G (Humboldt) | 22 |
| SF67 | JDF, J (Mendocino) | 52 | AET, M (Napa) | 20 | D (Humboldt) | 45 | E (Humboldt) | 14 |
| SF70 | HW, G (Humboldt) | 50 | EN, M (Napa) | 35 | F (Humboldt) | 74 | B (Del Norte) | 5 |
| SF74 | EN, M (Napa) | 48 | HW, G (Humboldt) | 19 | G (Humboldt) | 33 | B (Del Norte) | 8 |
| SF81 | RERE, N (Santa Clara) | 47 | EN, M (Napa) | 18 | C (Humboldt) | 38 | A (Del Norte) | 28 |
| SF73 | JDF, J (Mendocino) | 44 | HW, G (Humboldt) | 13 | B (Del Norte) | 14 | J (Mendocino) | 12 |
| B04 | AET, M (Napa) | 43 | HW, G (Humboldt) | 15 | H (Mendocino) | 19 | M (Napa) | 16 |
| CH1 | AET, M (Napa) | 43 | HW, G (Humboldt) | 15 | H (Mendocino) | 19 | M (Napa) | 16 |
| SF3 | AET, M (Napa) | 43 | HW, G (Humboldt) | 15 | H (Mendocino) | 19 | M (Napa) | 16 |
| SF63 | AET, M (Napa) | 43 | HW, G (Humboldt) | 15 | H (Mendocino) | 19 | M (Napa) | 16 |
| SF64 | AET, M (Napa) | 43 | HW, G (Humboldt) | 15 | H (Mendocino) | 19 | M (Napa) | 16 |
| SF76 | AET, M (Napa) | 43 | HW, G (Humboldt) | 15 | H (Mendocino) | 19 | M (Napa) | 16 |
| SF79 | AET, M (Napa) | 43 | HW, G (Humboldt) | 15 | H (Mendocino) | 19 | M (Napa) | 16 |
| SF82 | AET, M (Napa) | 43 | HW, G (Humboldt) | 15 | H (Mendocino) | 19 | M (Napa) | 16 |
| SF86 | AET, M (Napa) | 43 | HW, G (Humboldt) | 15 | H (Mendocino) | 19 | M (Napa) | 16 |
| SF87 | AET, M (Napa) | 43 | HW, G (Humboldt) | 15 | H (Mendocino) | 19 | M (Napa) | 16 |
| SF93 | AET, M (Napa) | 43 | HW, G (Humboldt) | 15 | H (Mendocino) | 19 | M (Napa) | 16 |
| BG120 | JDF, J (Mendocino) | 38 | HW, G (Humboldt) | 33 | F (Humboldt) | 29 | B (Del Norte) | 18 |
| SF84 | JDF, J (Mendocino) | 35 | DL (Napa) | 24 | J (Mendocino) | 30 | M (Napa) | 23 |
| SF91 | DL (Napa) | 33 | RERE, N (Santa Clara) | 32 | B (Del Norte) | 23 | N (Marine) | 18 |
| SF69 | JDF, J (Mendocino) | 31 | AET, M (Napa) | 31 | F (Humboldt) | 41 | H (Mendocino) | 15 |
| SF88 | JDF, J (Mendocino) | 31 | WEO, G (Humboldt) | 19 | L (Sonoma) | 25 | A (Del Norte) | 18 |
| SF90 | JDF, J (Mendocino) | 31 | WEO, G (Humboldt) | 19 | L (Sonoma) | 25 | A (Del Norte) | 18 |
| BA030549 | RERE, N (Santa Clara) | 28 | DL (Napa) | 24 | A (Del Norte) | 31 | D (Humboldt) | 9 |
| B30 | MtMa, O (Santa Clara) | 25 | RERI, O (Santa Clara) | 25 | O (Santa Cruz) | 24 | L (Sonoma) | 17 |
| B102 | LPF, M (Napa) | 21 | HW, G (Humboldt) | 17 | B (Del Norte) | 14 | L (Sonoma) | 12 |
| BG121 | JDF, J (Mendocino) | 21 | HW, G (Humboldt) | 17 | B (Del Norte) | 14 | L (Sonoma) | 12 |
| BG122 | JDF, J (Mendocino) | 21 | HW, G (Humboldt) | 17 | B (Del Norte) | 14 | L (Sonoma) | 12 |
| BG123 | JDF, J (Mendocino) | 21 | HW, G (Humboldt) | 17 | B (Del Norte) | 14 | L (Sonoma) | 12 |
| BG124 | JDF, J (Mendocino) | 21 | HW, G (Humboldt) | 17 | B (Del Norte) | 14 | L (Sonoma) | 12 |
| BG125 | JDF, J (Mendocino) | 21 | HW, G (Humboldt) | 17 | B (Del Norte) | 14 | L (Sonoma) | 12 |
| BG126 | JDF, J (Mendocino) | 21 | HW, G (Humboldt) | 17 | B (Del Norte) | 14 | L (Sonoma) | 12 |
| BG127 | JDF, J (Mendocino) | 21 | HW, G (Humboldt) | 17 | B (Del Norte) | 14 | L (Sonoma) | 12 |
| BG128 | JDF, J (Mendocino) | 21 | HW, G (Humboldt) | 17 | B (Del Norte) | 14 | L (Sonoma) | 12 |
| GOE_G | JDF, J (Mendocino) | 21 | HW, G (Humboldt) | 17 | B (Del Norte) | 14 | L (Sonoma) | 12 |
| GOE_K | JDF, J (Mendocino) | 21 | HW, G (Humboldt) | 17 | B (Del Norte) | 14 | L (Sonoma) | 12 |
| SF62 | JDF, J (Mendocino) | 21 | HW, G (Humboldt) | 17 | B (Del Norte) | 14 | L (Sonoma) | 12 |

Note: Highlighted by color are assignments in consensus with both reference data sets C and F (by yellow are northern and by blue are southern populations and watersheds, respectively).
